# Supplementary material for: PP1 and PP2A Use Opposite Phospho-dependencies to Control Distinct Processes at the Kinetochore
Source: Cell Rep. 2019 Aug 20;28(8):2206–2219.e8. doi: 10.1016/j.celrep.2019.07.067 (PMC6715587; doi:10.1016/j.celrep.2019.07.067)
Supplement: Document S1. Figures S1–S6 [file mmc1.pdf]

Cell Reports, Volume *28*

Supplemental Information

PP1 and PP2A Use Opposite Phospho-dependencies to Control Distinct Processes at the Kinetochore

Richard J. Smith, Marilia H. Cordeiro, Norman E. Davey, Giulia Vallardi, Andrea Ciliberto, Fridolin Gross, and Adrian T. Saurin

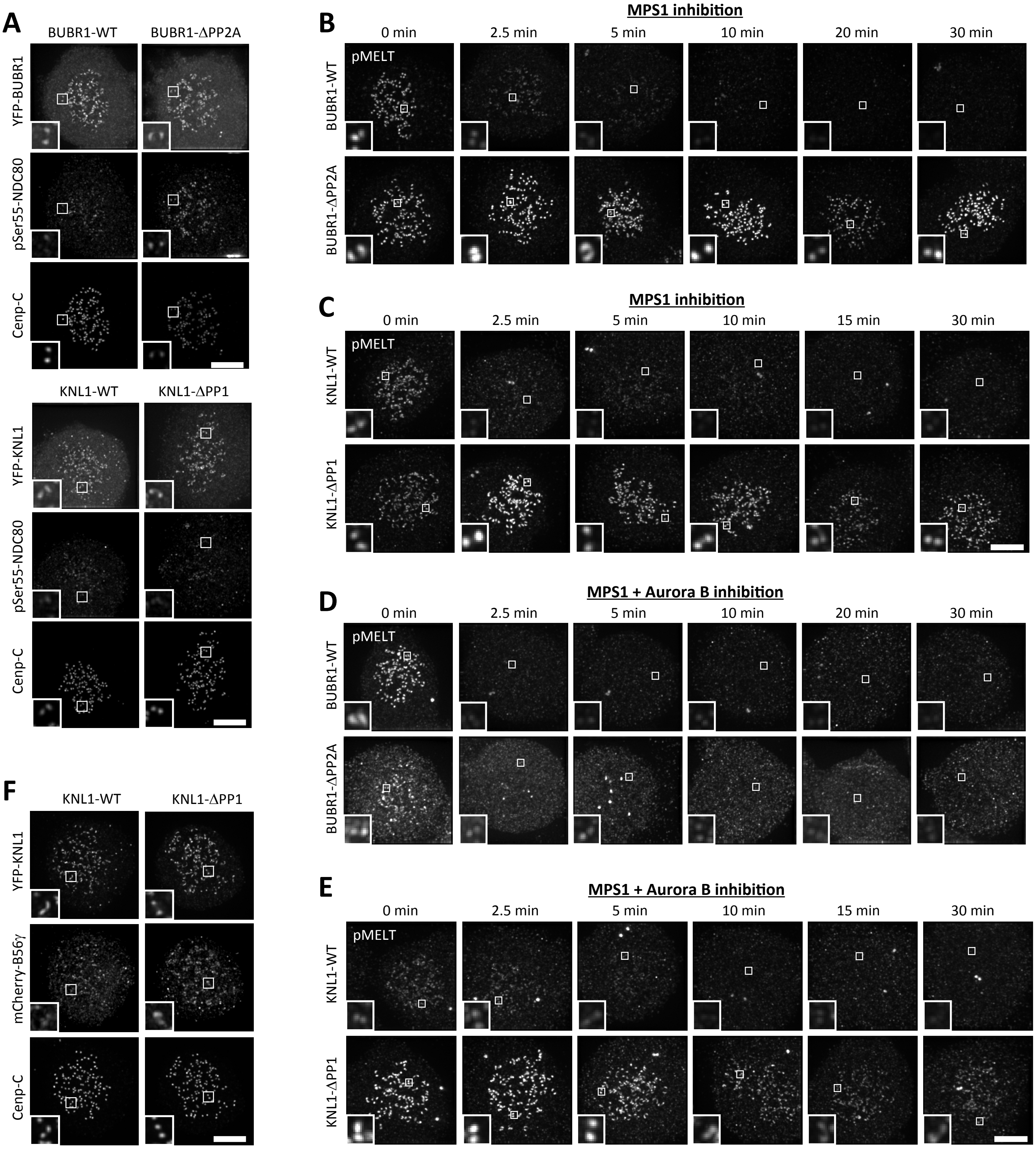

Figure S1. Immunofluorescence images to show that PP1-KNL1 and PP2A-B56 exert control over different pathways and processes at the kinetochore; related to figure 1. A-F. Example immunofluorescence images of the kinetochore quantifications shown in Figure 1B (A), 1C (B), 1D (C), 1E (D), 1F (E) and 1H (F). The images were chosen that most closely resemble the mean values in the quantifications. The insets show magnifications of the outlined regions. Scale bars = 5μm.

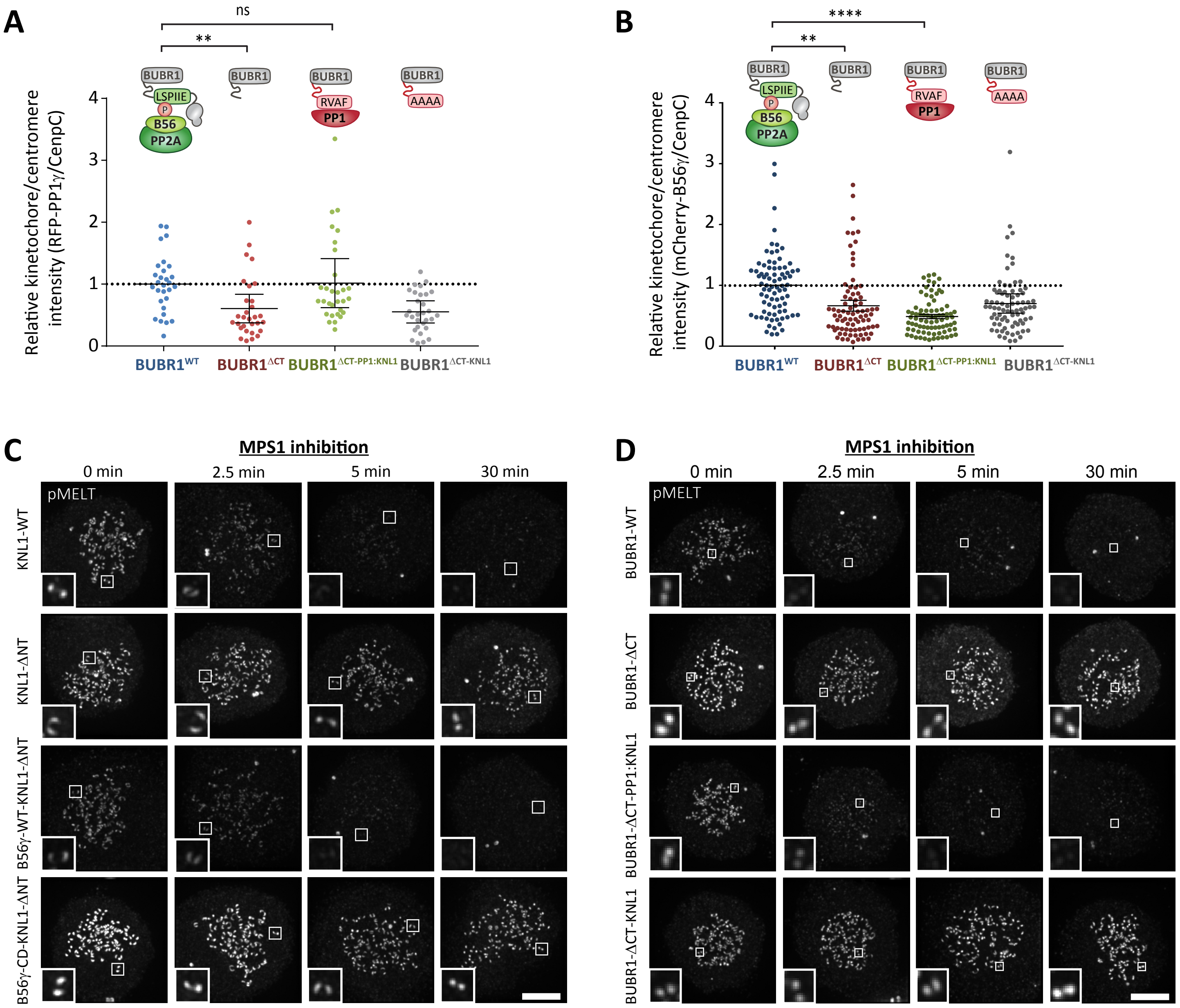

Figure S2. Quantification of kinetochore phosphatase levels and immunofluorescence images after swapping the phosphatase recruitment SLiMs between BUBR1 and KNL1; related to figure 2. A. RFP-PP1γ levels in the various mutant BUBR1 cells lines. Note that full removal of KNL1-PP1 from kinetochores only causes a 70% reduction in total kinetochore PP1γ (Nijenhuis et al., 2014). Graph shows 30 cells per condition from 3 experiments. **B.** Quantification of kinetochore mCherry-B56γ levels in the various mutant BUBR1 cells lines. Note that removal of the B56-SLiM on BUBR1 does not fully remove B56γ because a pool of B56γ remains bound to the centromere via Sgo1 (Vallardi et al., 2019) and this pool is also detected in our quantifications because this does not distinguish between kinetochores and centromeres. Graph shows 80 cells per condition from 4 experiments. For all kinetochore intensity graphs, each dot represents a cell and the errors bars display the variation between the experimental repeats (displayed as ±SD of the experimental means). **p<0.01, **** p<0.0001. **C-D.** Example immunofluorescence images of the kinetochore quantifications shown in Figure 2B (A) and 2D (B). The images were chosen that most closely resemble the mean values in the quantifications. The insets show magnifications of the outlined regions. Scale bars = 5µm.

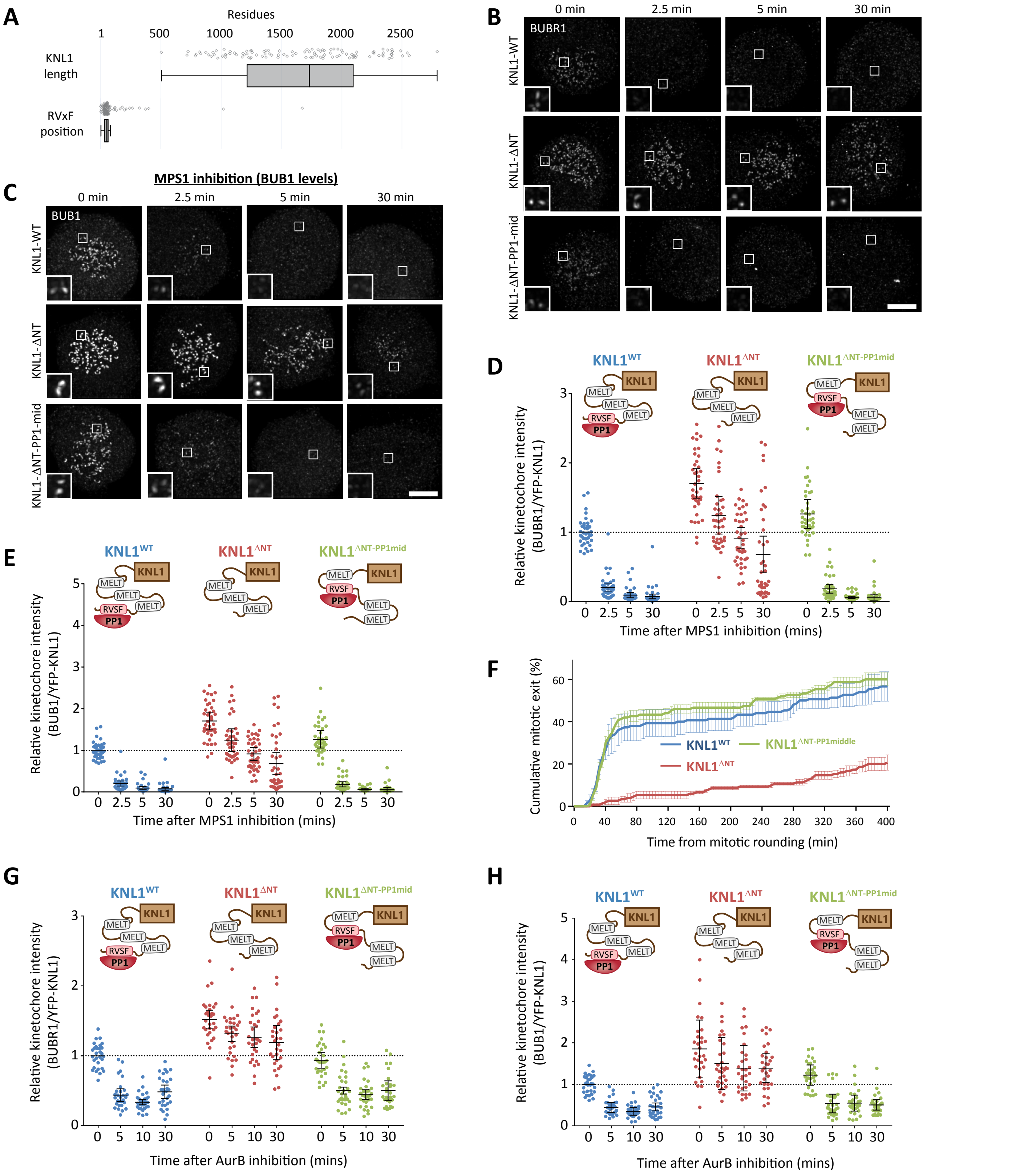

Figure S3. The PP1 binding SLiM is conserved at the N-terminus of KNL1 and this position is not critical for MELT dephosphorylation in nocodazole; related to figure 3. A. Position of first RVxF motif and total number of amino acids in 110 KNL1 orthologs from all eukaryotic supergroups; as identified in (Tromer et al., 2015). **B-E.** Example immunofluorescence images (B,C) and quantifications of kinetochore intensities (D,E) of BUBR1 (B,D) and BUB1 (C,E) from cells treated identically to those described in Figure 3C. **F.** Duration of mitotic arrest in cells treated with nocodazole and 2.5 μM AZ-3146. Graph shows the cumulative mean (±SEM) of 3 experiments, 50 cells per condition per experiment. **G,H.** Quantifications of kinetochore intensities of BUBR1 (G) and BUB1 (H) from nocodazole-arrested cells treated with the Aurora B inhibitor ZM-447439 (2 μM) for the indicated times. Kinetochore intensities graphs show 40 cells per condition from 4 experiments (D,E) or 30 cells from 3 experiments (G,H). MG132 was included to prevent Cyclin B degradation and mitotic exit following MPS1 inhibition. Each dot represents a cell and the errors bars display the variation between the experimental repeats (displayed as ±SD of the experimental means). The images were chosen that most closely resemble the mean values in the quantifications. The insets show magnifications of the outlined regions. Scale bars = 5μm.

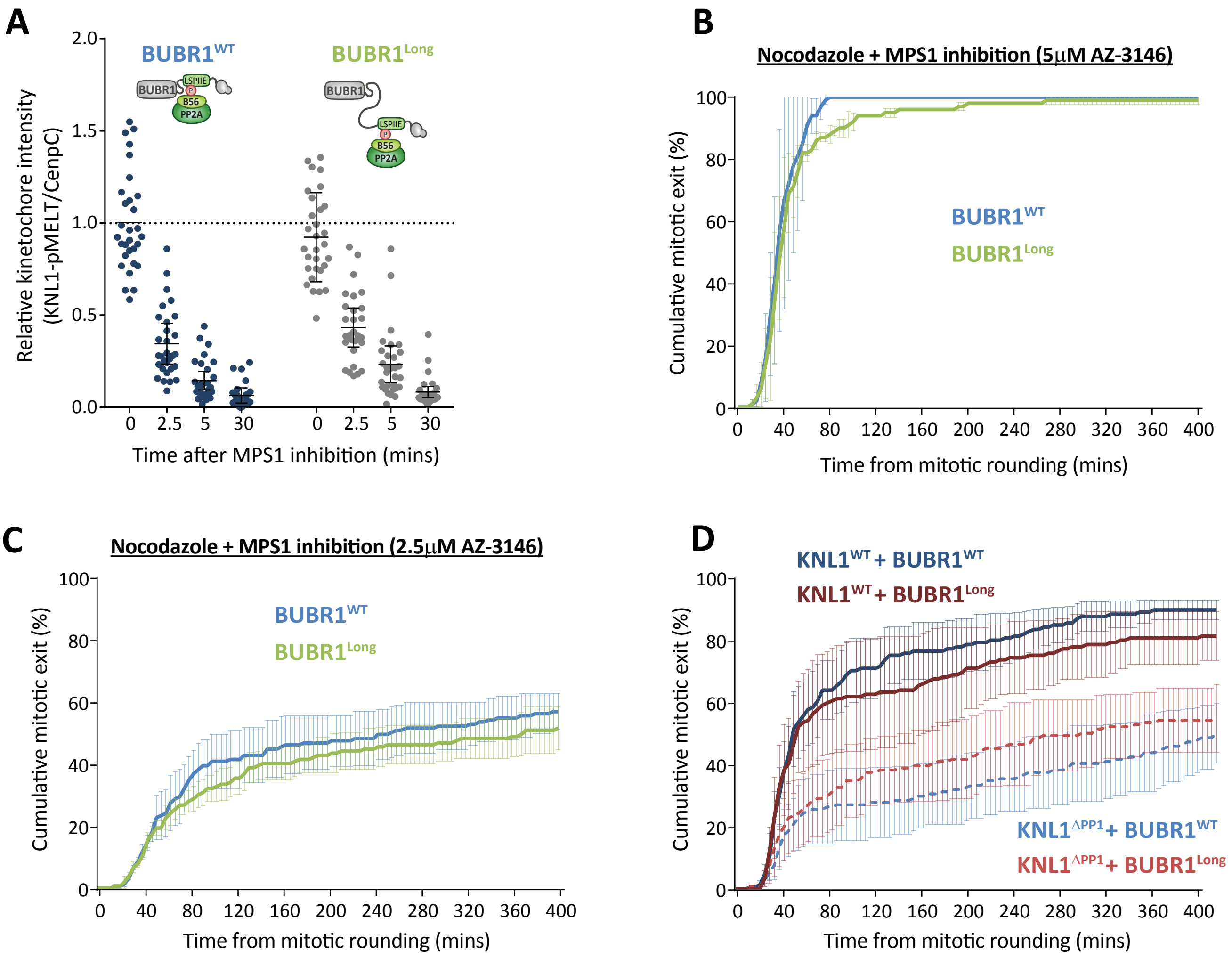

Figure S4. Insertion of a long flexible linker before the LxxIxE motif in BUBR1 does not improve the ability of PP2A to silence the SAC; related to figure 4. Effect of inserting a long flexible linker before the LxxIxE motif in BUBR1 on SAC phenotypes. **A.** KNL1-MELT dephosphorylation in nocodazole-arrested cells treated with 2.5 µM AZ-3146 for indicated times. MG132 was included in to prevent Cyclin B degradation and mitotic exit following MPS1 inhibition. Graph shows 30 cells per condition from 3 experiments. Each dot represents a cell and the errors bars display the variation between the experimental repeats (displayed as ±SD of the experimental means). **B-D.** Duration of mitotic arrest in cells treated with nocodazole and 5 µM (B) or 2.5 µM (C,D) AZ-3146. Experiments in D contain wild type and mutant combinations to remove PP1-KNL1 in the presence or absence of the flexible linker. Graph in B-D show the cumulative mean (±SEM) of 3 experiments, 50 cells per condition per experiment. 2 experiments from the BubR1-WT controls in D are also used in some experiments from Figure 3E (See Data File S2 for details).

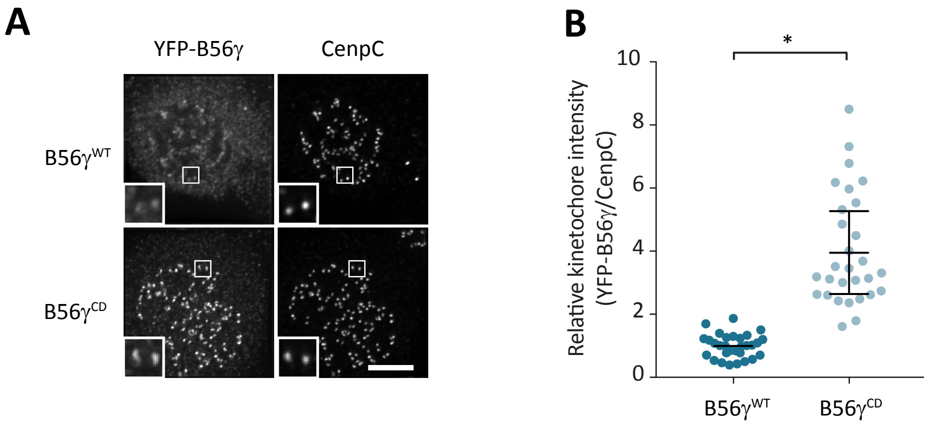

Figure S5. Effect of negative feedback on B56γ kinetochore levels; related to figure 5. A,B. Example immunofluorescence images (A) and quantification of kinetochore intensities (B) of YFP-B56γWT or YFP-B56γCD. The images were chosen that most closely resemble the mean values in the quantifications. The insets show magnifications of the outlined regions. Scale bars = 5μm. ns $p>0.05$, *$p<0.05$.

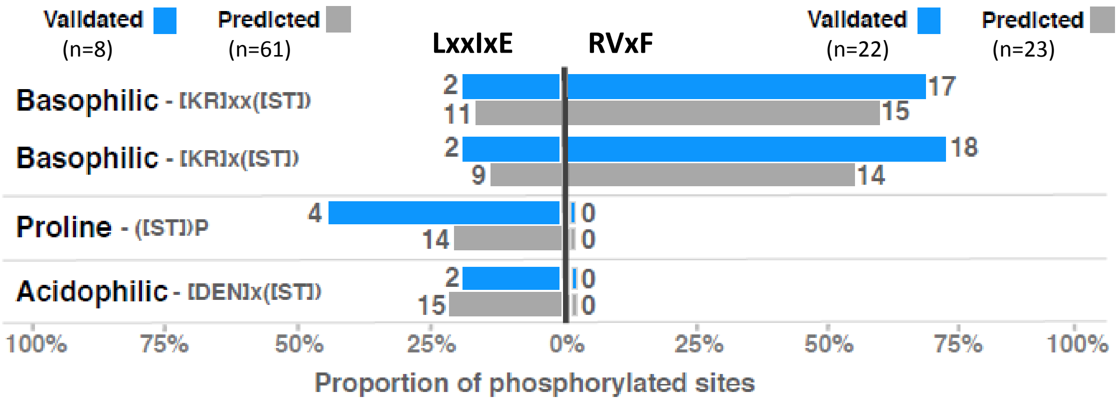

Figure S6. Summary of the kinase specificity determinants overlapping the phosphorylated and phosphorylatable sites in the experimentally validated LxxIxE and RVxF motifs; related to figure 6. The kinase specificity determinants of proline-directed, basophilic and acidophilic kinases matching the preferences of Cyclin dependent kinases, Aurora kinases and the Polo-like kinases, respectively. Note that Aurora phosphorylation of CDK1 sites is prevented, even if the correct basic sequences are present, because proline (+1) is a strong negative determinant within the Aurora consensus motifs (Alexander et al., 2011).
