# Supplementary material for: PP1 and PP2A Use Opposite Phospho-dependencies to Control Distinct Processes at the Kinetochore
Source: Cell Rep. 2019 Aug 20;28(8):2206–2219.e8. doi: 10.1016/j.celrep.2019.07.067 (PMC6715587; doi:10.1016/j.celrep.2019.07.067)
Supplement: Document S1. Article plus Supplemental Information [file mmc5.pdf]

Article

Cell Reports

PP1 and PP2A Use Opposite Phospho-dependencies to Control Distinct Processes at the Kinetochore

Graphical Abstract

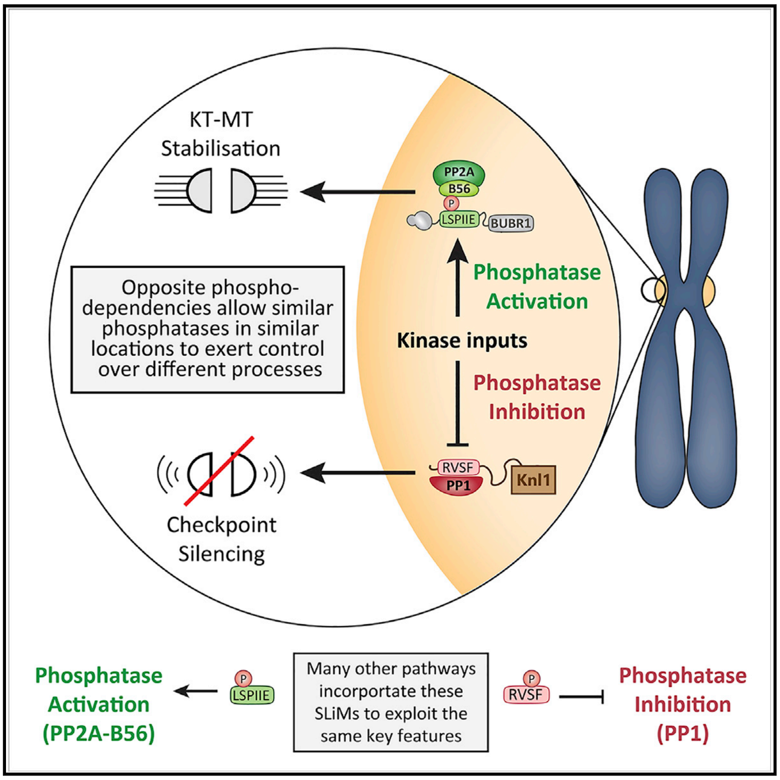

Authors

Richard J. Smith, Marilia H. Cordeiro, Norman E. Davey, Giulia Vallardi, Andrea Ciliberto, Fridolin Gross, Adrian T. Saurin

Correspondence

a.saurin@dundee.ac.uk

In Brief

Smith et al. investigate PP1 and PP2A-B56 specificity at kinetochores and conclude that the main difference between these phosphatases is their ability to respond in opposite ways to phosphorylation inputs. This allows them to produce distinct network behaviors and control different mitotic processes. These unique features have likely been exploited by many other signaling pathways throughout evolution.

Highlights

- PP1 and PP2A-B56 can functionally substitute for each other at the kinetochore
- The major difference is their ability to respond to phospho-inputs in opposite ways
- This underlies their distinct phenotypic behaviors
- Many other signaling pathways also select for these same key features

Smith et al., 2019, Cell Reports *28*, 2206–2219
August 20, 2019 © 2019 The Authors.
https://doi.org/10.1016/j.celrep.2019.07.067

CellPress

OPEN ACCESS
CellPress

Cell Reports
Article

PP1 and PP2A Use Opposite Phospho-dependencies to Control Distinct Processes at the Kinetochore

Richard J. Smith,[1,5] Marilia H. Cordeiro,[1,5] Norman E. Davey,[2,4] Giulia Vallardi,[1] Andrea Ciliberto,[3] Fridolin Gross,[3] and Adrian T. Saurin[1,6,*]
[1]Division of Cellular Medicine, School of Medicine, University of Dundee, Dundee DD1 9SY, UK
[2]Conway Institute of Biomolecular and Biomedical Research, University College Dublin, Belfield, Dublin 4, Ireland
[3]Istituto Firc di Oncologia Molecolare, IFOM, Milano, Italy
[4]Present address: Division of Cancer Biology, The Institute of Cancer Research, 237 Fulham Road, London SW3 6JB, UK
[5]These authors contributed equally
[6]Lead Contact
*Correspondence: a.saurin@dundee.ac.uk
https://doi.org/10.1016/j.celrep.2019.07.067

SUMMARY

PP1 and PP2A-B56 are major serine/threonine phosphatase families that achieve specificity by colocalizing with substrates. At the kinetochore, however, both phosphatases localize to an almost identical molecular space and yet they still manage to regulate unique pathways and processes. By switching or modulating the positions of PP1/PP2A-B56 at kinetochores, we show that their unique downstream effects are not due to either the identity of the phosphatase or its precise location. Instead, these phosphatases signal differently because their kinetochore recruitment can be either inhibited (PP1) or enhanced (PP2A) by phosphorylation inputs. Mathematical modeling explains how these inverse phospho-dependencies elicit unique forms of cross-regulation and feedback, which allows otherwise indistinguishable phosphatases to produce distinct network behaviors and control different mitotic processes. Furthermore, our genome-wide analysis suggests that these major phosphatase families may have evolved to respond to phosphorylation inputs in opposite ways because many other PP1 and PP2A-B56-binding motifs are also phospho-regulated.

INTRODUCTION

Protein phosphatase 1 (PP1) and protein phosphatase 2A (PP2A) are large phosphatase families that are responsible for most of the serine/threonine dephosphorylation in eukaryotic cells (Brautigan and Shenolikar, 2018; Heroes et al., 2013). This is exemplified by the fact that PP2A inhibition causes approximately half of the phosphorylation sites in the human proteome to change significantly (Kauko et al., 2018). PP1 and PP2A use structurally related catalytic domains that are remarkably well-conserved and relatively promiscuous *in vitro* (Ingebritsen and Cohen, 1983). *In vivo*, however, they are believed to achieve specificity by interacting with short linear motifs (SLiMs) that localize them to their required sites of action (Brautigan and Shenolikar, 2018). The best-characterized SLiM for PP1 is the RVxF motif, which is present in approximately 90% of the validated PP1-interacting proteins (Heroes et al., 2013). The best-characterized SLiM for PP2A is the LxxIxE motif, which binds to the regulatory subunit of the PP2A-B56 holoenzyme complex (Hertz et al., 2016).

This simplistic model of co-localization driving function explains nicely how these phosphatases can target specific substrates, but it does not explain why these substrates select to interact specifically with one phosphatase over the other when their catalytic activities are apparently very similar. In that sense, it fails to capture the essence of why PP1 and PP2A have evolved to regulate different signals. They must presumably possess specific features that are repeatedly selected for by different pathways throughout the course of evolution, although exactly what these features are still remains unclear. It is important to address this because it may help to reveal why these two major phosphatase families have evolved to fulfil different signaling roles.

One major distinction between PP1 and PP2A is their ability to be regulated differently. This can occur directly on the holoenzymes; for example, via catalytic subunit phosphorylation or the binding of catalytic inhibitors (Verbinnen et al., 2017; Rogers et al., 2016; Grallert et al., 2015). A well-studied example of this is the inhibition of PP2A-B55 during mitosis by the ARPP19/ENSA phospho-proteins (Gharbi-Ayachi et al., 2010; Mochida et al., 2010). Although direct regulation of the holoenzyme is useful for modulating global phosphatase activity, there are many situations when individual pathways or substrates must be regulated separately. In these cases, the regulation can occur directly on the SLiMs within these pathways that are needed to direct the phosphatases toward specific substrates. Interestingly, in this respect, PP1 and PP2A-B56 behave in opposite ways; PP1 binding to the RVxF motif can be repressed by phosphorylation (Kim et al., 2003; Nasa et al., 2018), whereas PP2A-B56 interaction with the LxxIxE motif can be enhanced by phosphorylation (Hertz et al., 2016; Wang et al., 2016a, 2016b). These unique modes of phospho-regulation could allow PP1 and PP2A-B56 to perform very different signaling roles; however, it is difficult to dissociate

 © 2019 The Authors.
This is an open access article under the CC BY license (http://creativecommons.org/licenses/by/4.0/).

whether it is these properties or others, such as catalytic preferences, that are more important in any given situation.

To investigate this further, we chose to focus on the kinetochore, which is a multi-complex structure assembled on chromosomes during mitosis to mediate their attachment to microtubules. Although this complex contains over 100 different proteins, PP1 and PP2A-B56 are recruited via their SLiMs to the same molecular scaffold, KNL1, to regulate kinetochore-microtubule attachments and the spindle assembly checkpoint (SAC) (Saurin, 2018). These processes are critical for genome stability because microtubules bind to kinetochores to segregate the duplicated chromosomes equally, and the SAC holds the mitotic state to give time for these microtubules to attach correctly. Importantly, even though PP1 and PP2A are recruited to a very similar molecular space on kinetochores, they still appear to control these key mitotic processes differently, as evidenced by the fact that removing either phosphatase produces markedly distinct phenotypic effects (these will be discussed in detail below) (Saurin, 2018). It is currently unclear how these phosphatases achieve specificity in such a crowded molecular environment or, indeed, why they are both needed to carry out different roles at the kinetochore. We therefore rationalized that this would be an ideal system to reveal answers about phosphatase specificity and functional diversity within the broader signaling context.

Using the direct approach of switching the phosphatases or their SLiMs at the kinetochore, we demonstrate that their unique phenotypic effects cannot be explained by either catalytic preferences or positional differences. Instead, we demonstrate that phenotypic diversity arises because the phosphatases are recruited via SLiMs that display opposite phospho-dependencies and, as a result, are subject to different forms of cross-regulation and feedback. Therefore, this study explains how downstream "specificities" can depend entirely on the mode of upstream regulation, and it establishes a paradigm to explain how these two major phosphatase families may have evolved to couple to phosphorylation inputs in opposite ways.

RESULTS

PP1-KNL1 and PP2A-B56 Exert Control over Different Kinetochore Processes

KNL1 is a key signaling scaffold that functions at kinetochores to generate the SAC signal and regulate the attachment of microtubules. Critical for both of these processes are the "MELT" motifs (for the consensus sequence Met-Glu-Leu-Thr) that are scattered along the N-terminal half of KNL1 and phosphorylated by MPS1 kinase to recruit the BUB1/BUBR1/BUB3 complex (London et al., 2012; Shepperd et al., 2012; Yamagishi et al., 2012; Overlack et al., 2015; Primorac et al., 2013; Zhang et al., 2014; Vleugel et al., 2013). This complex has two main functions: (1) it modulates Aurora B activity to regulate kinetochore-microtubule attachments (Aurora B is a kinase that can phosphorylate kinetochores to detach microtubule fibers; Krenn and Musacchio, 2015), and (2) it provides a platform to recruit all other proteins needed for the SAC to delay mitotic exit (Saurin, 2018). Crucially, both of these functions are regulated by two co-localized phosphatase complexes: PP1, which is bound to the SILK and RVSF motifs in the N terminus of KNL1 (PP1-KNL1; note that Aurora B phosphorylates these motifs to inhibit PP1 binding; Liu et al., 2010; Espeut et al., 2012; Meadows et al., 2011; Nijenhuis et al., 2014; Rosenberg et al., 2011), and PP2A-B56, which binds to an LSPIIE motif in BUBR1 (note that CDK1 and PLK1 both phosphorylate this motif to enhance PP2A-B56 interaction; Foley et al., 2011; Kruse et al., 2013; Suijkerbuijk et al., 2012; Xu et al., 2013; Nijenhuis et al., 2014; Espert et al., 2014). There has been debate surrounding exactly which phosphatase controls which process (Saurin, 2018); therefore, we begin by carefully dissecting their individual roles at the kinetochore.

As shown previously by others (Shrestha et al., 2017), removal of the PP2A-B56 SLiM in BUBR1 (BUBR1$^{\Delta PP2A}$) causes severe defects in chromosome alignment whereas inactivation of the PP1 SLiM in KNL1 (KNL1$^{\Delta PP1}$) does not (Figure 1A; note that, in these and all subsequent experiments, siRNA-mediated gene knockdown was used in combination with doxycycline-inducible replacement of the mutant gene from an FRT locus; STAR Methods). Interestingly, we observed that these defects are associated with enhanced phosphorylation of the NDC80 tail region (Figures 1B and S1A), a key Aurora B substrate that must be dephosphorylated to stabilize kinetochore-microtubule attachments (Krenn and Musacchio, 2015). In contrast to these differential effects on chromosome alignment, PP1-KNL1 and PP2A-B56 are both needed to allow KNL1 MELT dephosphorylation and SAC silencing following inhibition of the upstream kinase MPS1 (Figures 1C, 1D, S1B, and S1C; Espert et al., 2014; Nijenhuis et al., 2014). However, even in this situation, the BUBR1$^{\Delta PP2A}$ and KNL1$^{\Delta PP1}$ phenotypes differ because the effects of PP2A-B56 loss can be specifically rescued by Aurora B inhibition (Figures 1E, 1F, S1D, and S1E; note that this is not due to differential effects on microtubule attachments because all SAC assays were performed in nocodazole to depolymerize microtubules). We hypothesized previously that PP2A-B56 sits upstream of PP1 in SAC silencing by suppressing Aurora B-mediated phosphorylation of KNL1 to allow PP1-KNL1 association (Nijenhuis et al., 2014). This is consistent with the results shown in Figure 1G, which show that mutating these Aurora B sites in KNL1 (KNL1$^{PP1(2A)}$) allows nocodazole-arrested BUBR1$^{\Delta PP2A}$ cells to exit mitosis rapidly following MPS1 inhibition. Therefore, rescuing PP1-KNL1 can bypass the requirement for PP2A-B56 in SAC silencing. Importantly, the same is not true in reverse because PP2A-B56 is present on kinetochores in KNL1$^{\Delta PP1}$ cells (Figures 1H and S1F), and yet these cells can still not silence the SAC (Figure 1D; Nijenhuis et al., 2014).

In summary, PP1 and PP2A-B56 are recruited via their respective SLiMs to a very similar molecular space on kinetochores and yet they still manage to exert control over different substrates and different mitotic processes. PP2A-B56 antagonizes Aurora B signals to regulate kinetochore-microtubule attachments and PP1-KNL1 interaction, whereas PP1-KNL1 antagonizes MPS1 signals to silence the SAC (Figure 1I). PP2A-B56 may also contribute to SAC silencing directly, but it cannot support MELT dephosphorylation without PP1-KNL1 (Figures 1D and 1H). Similarly, PP1-KNL1 could help to stabilize initial

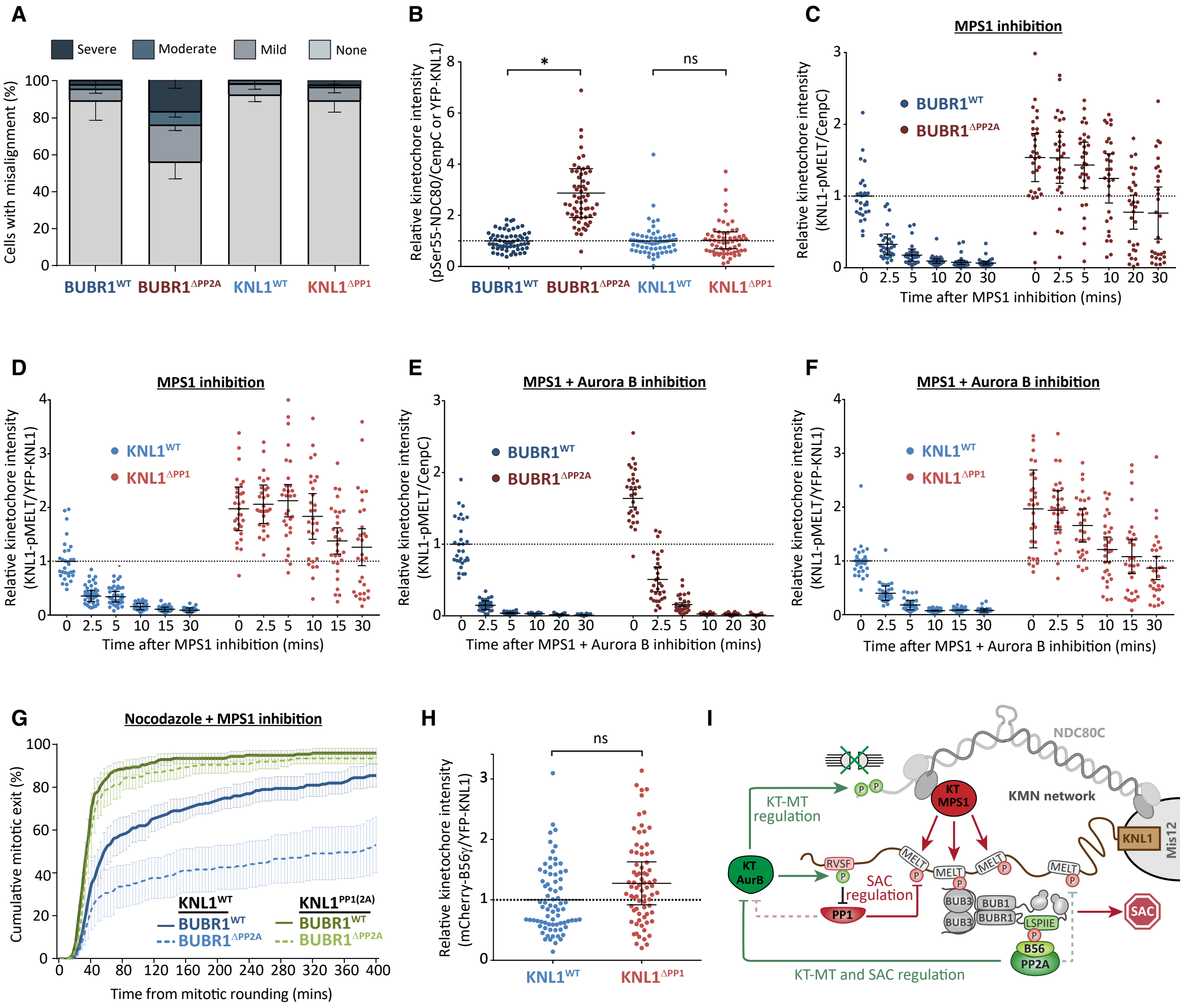

Figure 1. PP1-KNL1 and PP2A-B56 Exert Control over Different Pathways and Processes at the Kinetochore

(A and B) Effect of phosphatase-binding mutants on chromosomal alignment (A) and pSer55-NDC80 kinetochore levels in nocodazole (B). The graph in (A) shows the mean (−SD) of 3 experiments, at least 100 cells per condition per experiment. The graph in (B) shows data from 60 cells per condition from 4 experiments.

(C–F) Effect of phosphatase-binding mutants on KNL1-MELT dephosphorylation in nocodazole-arrested cells treated with the MPS1 inhibitor AZ-3146 (2.5 μM) for the indicated times, either alone (C and D) or in combination with the Aurora B inhibitor ZM-447439 (2 μM, E and F). MG132 was included in all treatments to prevent Cyclin B degradation and mitotic exit following MPS1 inhibition. The graphs in C–E include 30 cells per condition from 3 experiments.

(G) Duration of mitotic arrest in cells expressing various WT and phosphatase-binding mutant combinations and treated with nocodazole and 2.5 μM AZ-3146. The graph shows the cumulative mean (±SEM) of 4 experiments, 50 cells per condition per experiment.

(H) Kinetochore B56γ levels in nocodazole-arrested cells expressing WT or PP1 binding-deficient KNL1 (KNL1$^{\Delta PP1}$). The graph shows 75 cells per condition from 4 experiments.

(I) A schematic model depicting the primary effects of PP1 and PP2A-B56 on the KMN network. PP2A-B56 is shown to regulate Aurora B directly, but this is simply meant to represent co-antagonism of both Aurora B substrates at the kinetochore (pRVSF and pNDC80). KT, kinetochore; MT, microtubule.

For all kinetochore intensity graphs, each dot represents a cell, and the error bars display the variation between the experimental repeats (displayed as ± SD of the experimental means). Not significant, ns, $p > 0.05$; *$p < 0.05$.

microtubule attachments, but it is not essential, and it is also not able to support proper chromosome alignment in the absence of PP2A-B56 (Figure 1A). Therefore, both of these potential links are still included in the model shown in Figure 1I, but only as dotted arrows.

PP1 and PP2A-B56 Can Functionally Substitute for Each Other at Kinetochores

The simplest explanation for the observed phenotypic differences is that PP1 and PP2A are different catalytic enzymes that could produce specific effects at the kinetochore. Therefore,

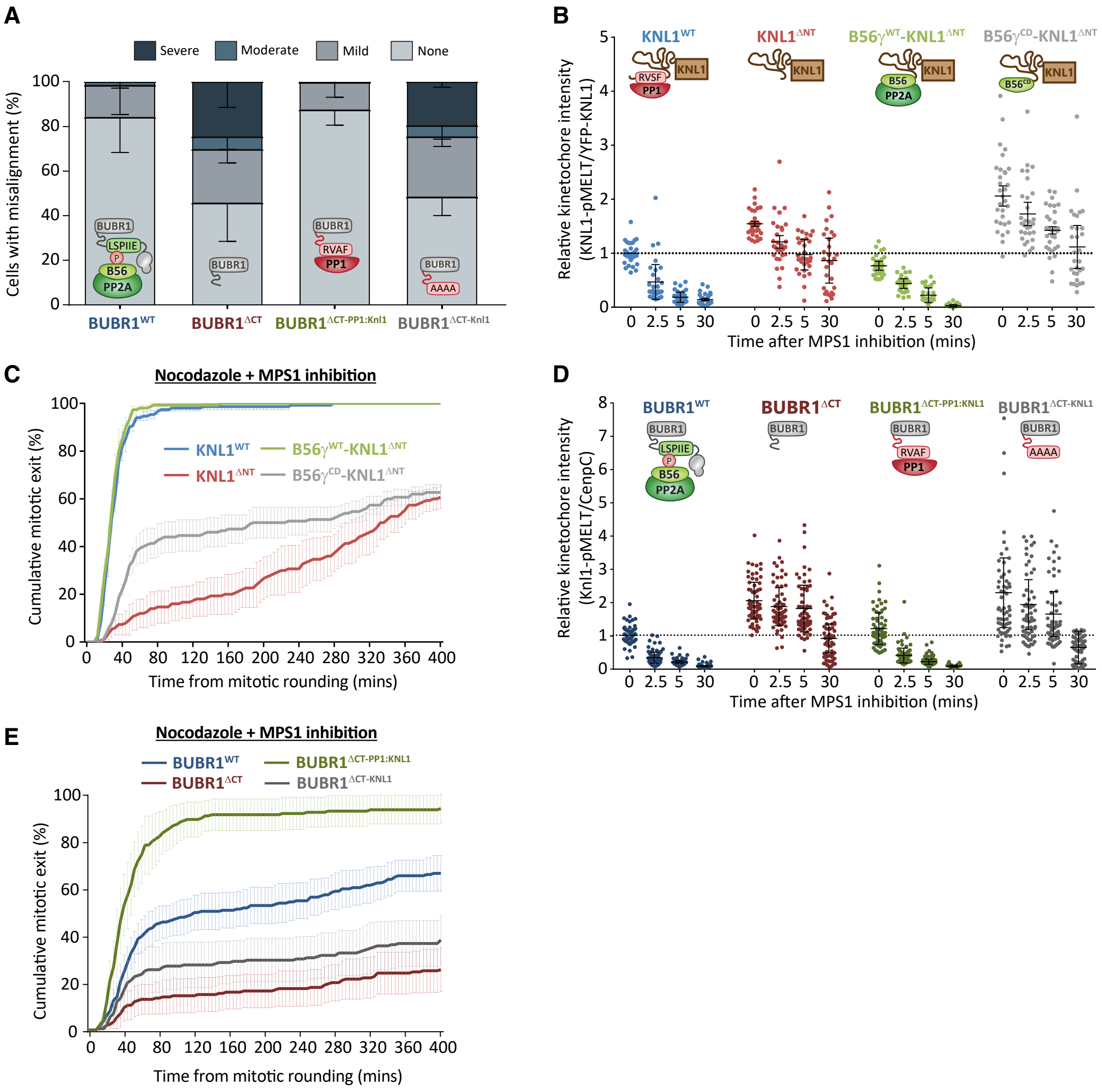

Figure 2. PP1 and PP2A-B56 Can Functionally Substitute for Each Other at the Kinetochore

(A) Effect of altering the phosphatase at BUBR1 on chromosomal alignment. The graph shows the mean (−SD) of 3 experiments, with at least 100 cells per condition per experiment.

(B and C) Effect of altering the phosphatase at KNL1 on SAC phenotypes.

(B) KNL1-MELT dephosphorylation in nocodazole-arrested cells treated with the MPS1 inhibitor AZ-3146 (2.5 μM) for the indicated times. The graph shows 30 cells per condition from 3 experiments.

(C) Duration of mitotic arrest in cells treated with nocodazole and 5 μM AZ-3146. The graph shows the cumulative mean (±SEM) of 3 experiments, 50 cells per condition per experiment.

(D and E) Effect of altering the phosphatase at BUBR1 on SAC phenotypes.

(D) KNL1-MELT dephosphorylation in nocodazole-arrested cells treated with the MPS1 inhibitor AZ-3146 (2.5 μM) for the indicated times. The graph shows 50–60 cells per condition from 6 experiments.

(E) Duration of mitotic arrest in cells treated with nocodazole and 2.5 μM AZ-3146. The graph shows the cumulative mean (±SEM) of 4 experiments, 50 cells per condition per experiment.

MG132 was included in treatments in (B) and (D) to prevent Cyclin B degradation and mitotic exit following MPS1 inhibition. For all kinetochore intensity graphs, each dot represents a cell, and the errors bars display the variation between the experimental repeats (displayed as ± SD of the experimental means).

to test whether the identity of the phosphatase is a key determinant of its function, we deleted the SLiMs that recruit PP1 or PP2A-B56 to kinetochores and asked whether the resulting phenotypes could be rescued when the alternative phosphatase is recruited in its place. Figure 2A shows that the chromosome alignment defects following truncation of BUBR1 before the PP2A-B56 binding region (BUBR1$^{\Delta CT}$) can be rescued when a short region from the KNL1 N terminus is fused in its place to

recruit PP1 (BUBR1$^{\Delta CT\text{-}PP1:KNL1}$; note that the Aurora B sites are mutated in the SLiMs to prevent Aurora B from inhibiting PP1 binding). This is dependent on PP1 binding because the effect is lost when the N-terminal fusion has the PP1-binding SLiM mutated (BUBR1$^{\Delta CT\text{-}KNL1}$) to prevent PP1 recruitment (Figure 2A; see Figure S2A and S2B for a demonstration of phosphatase switching under these conditions). Conversely, if the first 70 amino acids of the KNL1 N terminus are removed, which contains the PP1 binding region (KNL1$^{\Delta NT}$), then SAC silencing and MELT dephosphorylation are inhibited following MPS1 inhibition (Figures 2B, 2C, and S2C). However, if B56 is tethered directly to the N terminus of KNL1 (B56γ-KNL1$^{\Delta NT}$), then both of these effects can be fully rescued (Figures 2B, 2C, and S2C). This requires PP2A catalytic activity because fusion of a B56 mutant that cannot bind the catalytic domain (B56γ^{CD}-KNL1$^{\Delta NT}$; Vallardi et al., 2019) does not support SAC silencing. Finally, preventing PP2A-B56 recruitment to BUBR1 also gives a SAC silencing defect, and this can also be fully rescued by recruiting PP1 in its place (Figures 2D, 2E, and S2D). Therefore, both phosphatases can functionally substitute for each other when their respective positions at the kinetochore are switched. This demonstrates that the phenotypic differences cannot be explained by the identity of the individual phosphatases.

PP1 and PP2A-B56 Can Function from Different Positions at the KMN Network

If identity is not important for function, then the precise positions may be critical instead. For example, although PP1 or PP2A are recruited to the same molecular subcomplex on kinetochores, they may only have restricted access to a subset of different substrates from their exact positions on KNL1 and BUBR1. To address this, we first focused on kinetochore-microtubule attachment regulation because this was clearly defective when phosphatases were absent from the BUBR1 position (Figures 1A and 2A). Importantly, however, this position does not appear to be critical because chromosomal alignment defects in BUBR1$^{\Delta PP2A}$ cells could be rescued when B56 was recruited to the N terminus of KNL1 instead (B56γ-KNL1$^{\Delta NT}$) (Figure 3A). Therefore, PP2A-B56 can support chromosomal alignment from either the BUBR1 or KNL1 position.

We next examined why PP1-KNL1 was sufficient on its own to support SAC silencing whereas PP2A-B56 bound to BUBR1 was not (Figures 1C–1H). The SLiM that recruits PP1 is conserved at the N terminus of KNL1 throughout evolution (Figures 3B and S3A); therefore, we hypothesized that this position may be critical to access the MELTs. Surprisingly, however, moving the PP1-binding SLiM into the middle of KNL1 (KNL1$^{\Delta NT\text{-}PP1mid}$) had little effect on MELT dephosphorylation (Figure 3C), Bub complex removal from kinetochores (Figures S3B–S3E), or SAC silencing (Figures 3D and S3F) following MPS1 inhibition in nocodazole. Neither did it affect the ability of Aurora B to regulate the recruitment of BUB1/BUBR1 to KNL1 (Figures S3G and S3H). Therefore, PP1 does not need to be positioned at the N terminus of KNL1 to silence the SAC in the absence of microtubules. Although the exact position does not appear to be important, the phosphatase could still require a unique feature of KNL1 itself to silence the SAC, such as its predicted flexibility. This might explain why PP2A-B56 bound to BUBR1 could not dephosphorylate the MELT motifs in KNL1$^{\Delta PP1}$ cells (Figures 1D and 1H). Although we had already observed that PP1 can silence the SAC when bound to BUBR1 (Figures 2D and 2E), this effect might be direct or indirect because PP1 could simply dephosphorylate the SILK/RVSF motifs to recruit an additional PP1 molecule to KNL1. To distinguish between these possibilities, we created double-mutant cells in which the BUBR1 phosphatase could be switched in either the presence or absence of PP1-KNL1. Importantly, SAC silencing was still rescued in KNL1$^{\Delta PP1}$ cells by a BUBR1 mutant that recruits PP1 even though it could not be recovered in the same cells by a BUBR1 wild type (WT) that can bind to PP2A-B56 (Figure 3E). Therefore, PP1 can silence the SAC directly when positioned at either KNL1 or BUBR1.

In summary, PP1 and PP2A-B56 have specific functions at the kinetochore (Figure 1), but these cannot be explained by differences in either their catalytic subunits (Figure 2) or their spatial positioning (Figure 3). This is surprising because these are thought to be the principal determinants of phosphatase specificity, and if these phosphatases do not display any obvious specificity, then it is not easy to rationalize why they produce differential effects at the kinetochore. Furthermore, if the identity of the phosphatase is not important, as Figure 2 demonstrates, then it is not clear why there is such a difference in the ability of PP1 or PP2A to support kinetochore-microtubule attachment from the KNL1 N terminus (compare both BUBR1$^{\Delta PP2A}$ conditions in Figure 3A) and SAC silencing from the BUBR1 position (compare both KNL1$^{\Delta PP1}$ conditions in Figure 3E). However, as well as switching phosphatases in these key experiments, we also abolished their regulation by phosphorylation inputs. In particular, Aurora B phosphorylates the KNL1-SLiMs to inhibit PP1 (Liu et al., 2010), but when microtubule attachments were rescued by recruitment of PP2A-B56 to KNL1, we directly tethered B56 and lost these regulatory inputs (Figure 3A). In addition, CDK1 and PLK1 phosphorylate the BUBR1-SLiM to recruit PP2A-B56 (Huang et al., 2008; Elowe et al., 2007; Kruse et al., 2013; Suijkerbuijk et al., 2012; Wang et al., 2016a, 2016b), but when the SAC was rescued by recruiting PP1 to BUBR1, we removed this phospho-dependence (Figure 3E). Therefore, we rationalized that it may be the unique forms of SLiM regulation that prevent PP1-KNL1 from stabilizing microtubule attachments when PP2A-B56 is removed and restrict PP2A-B56 from silencing the SAC when PP1-KNL1 is absent.

PP1-KNL1 and PP2A-B56 Use Opposite Phospho-Dependencies to Control Distinct Kinetochore Processes

A major difference in their SLiM regulation is that phosphorylation of KNL1 represses PP1 binding, whereas phosphorylation of BUBR1 enhances PP2A-B56 binding. Therefore, even when these phosphatases display no downstream specificity at kinetochores, removal of their SLiMs will enhance phosphorylation of the opposing SLiM and produce opposite effects on phosphatase localization (Figure 4A). Indeed, inhibiting BUBR1:PP2A-B56 interaction is known to enhance Aurora B-mediated phosphorylation of the KNL1 SLiM to prevent PP1 binding (Nijenhuis et al., 2014). This is not a specific effect of PP2A-B56 per se because removal of PP2A-B56 from BUBR1 enhances

OPEN ACCESS
CellPress

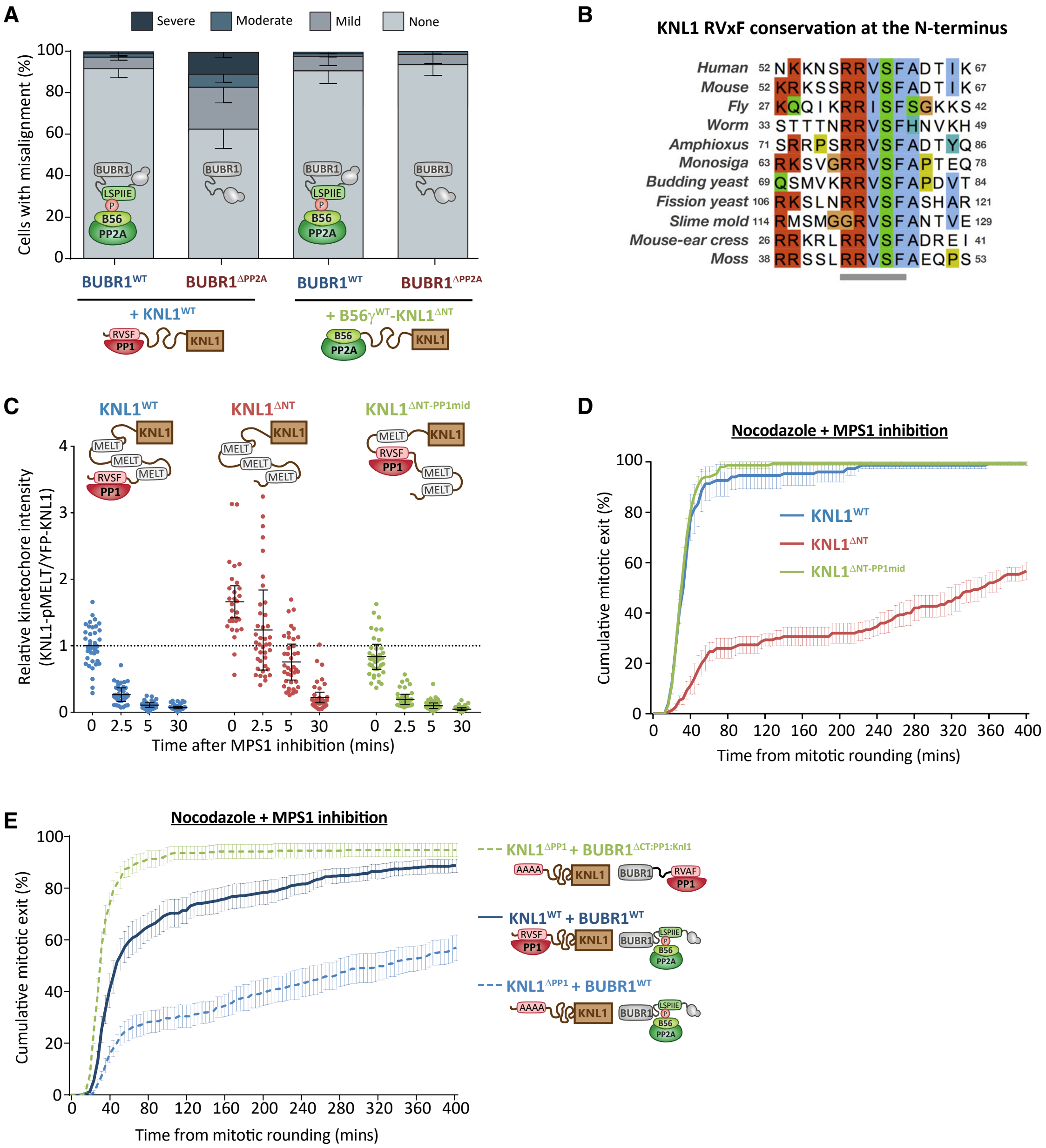

Figure 3. The Exact Positions of PP1 and PP2A-B56 Are Not Critical for Their Kinetochore Functions

(A) Chromosomal alignment after removing PP2A-B56 from BUBR1 and repositioning it at KNL1. The graph shows the mean (−SD) of 3 experiments, at least 100 cells per condition per experiment.

(B) Conservation of the RVxF SLiM at the N terminus of KNL1. Sequences are colored using the ClustalW scheme.

(C and D) Effect of relocating the PP1 SLiMs to the middle of KNL1 on SAC phenotypes.

(C) KNL1-MELT dephosphorylation in nocodazole-arrested cells treated with the MPS1 inhibitor AZ-3146 (5 μM) for the indicated times. The graph shows 30–40 cells per condition from 4 experiments. MG132 was included to prevent Cyclin B degradation and mitotic exit following MPS1 inhibition. Each dot represents a cell, and the errors bars display the variation between the experimental repeats (displayed as ± SD of the experimental means).

(D) Duration of mitotic arrest in cells treated with nocodazole and 5 μM AZ-3146.

(E) Effect of switching PP1 from KNL1 to BUBR1 on SAC silencing. Shown is the duration of mitotic arrest in cells expressing different WT and mutant combinations and treated with nocodazole and 2.5 μM AZ-3146.

The graphs in (D) and (E) show the cumulative mean (±SEM) of 3 experiments (D) or 8 experiments (E), 50 cells per condition per experiment. ns, $p > 0.05$; *$p < 0.05$.

KNL1-RVSF phosphorylation, and this can be rescued by recruiting PP1 to BUBR1 instead (Figure 4B). Therefore, inhibiting phosphatase activity at BUBR1 also inhibits it at KNL1 because the PP1:SLiM interaction is repressed by phosphorylation. Importantly, if these phosphorylation sites are mutated to alanine to rescue PP1-KNL1 in BUBR1$^{\Delta PP2A}$ cells (KNL1$^{PP1(2A)}$), then

OPEN ACCESS
CellPress

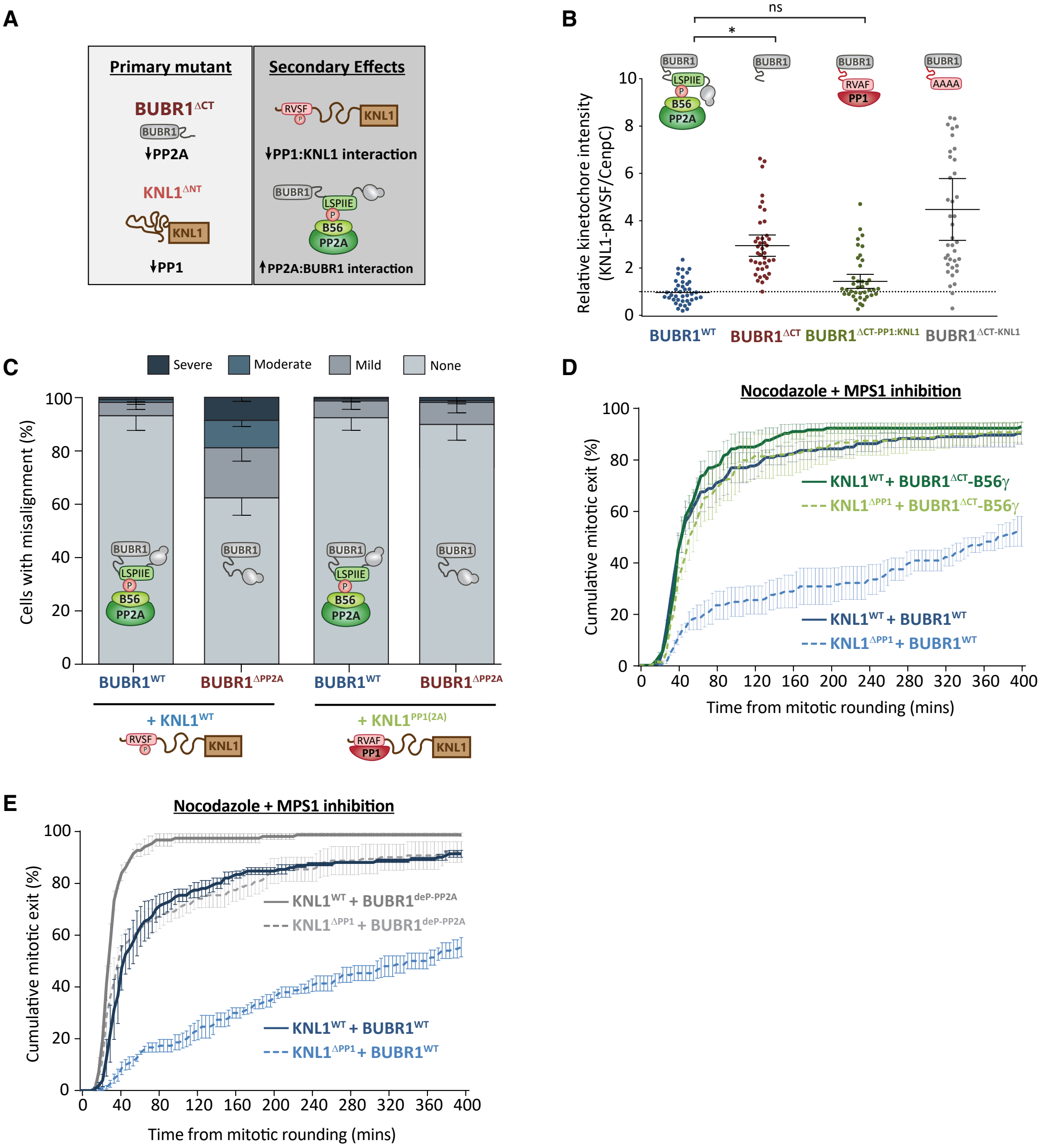

Figure 4. PP1-KNL1 and PP2A-B56 Use Opposite Phospho-dependencies to Control Distinct Processes at the Kinetochore

(A) Schematic to illustrate how cross-regulation between SLiMs affects kinetochore phosphatase levels.

(B) Effect of altering the phosphatase at BUBR1 on KNL1-pRVSF kinetochore levels in nocodazole. The graph shows data from 40 cells per condition from 4 experiments. Each dot represents a cell, and the error bars display the variation between the experimental repeats (displayed as ± SD of the experimental means).

(C) Chromosome alignment in cells expressing mutant combinations to prevent phosphorylation of the PP1 SLiMs following removal of PP2A-B56 from BUBR1. The graph shows the mean (−SD) of 3 experiments, at least 100 cells per condition per experiment.

(D and E) Effect of removing the phospho-dependence of PP2A-B56 on SAC silencing by either direct BUBR1-B56γ fusion (D) or mutation of the PP2A-binding SLiM in BUBR1 (E). Shown is the duration of mitotic arrest in cells expressing various WT and mutant combinations and treated with nocodazole and 2.5 μM AZ-3146. The graphs show the cumulative mean (±SEM) of 3 experiments, 38–50 cells per condition per experiment. The BubR1-WT controls in (D) were also used in some experiments shown in Figure 3E (see Data S2 for details).

chromosome alignment defects are also recovered (Figure 4C). Therefore, either phosphatase in either position can support chromosomal alignment (Figures 2A, 3A, and 4C). There appears to be a specific role for PP2A-B56 because when it is removed from BUBR1, then PP1-KNL1 is also lost (assuming that Aurora B is active on kinetochores to phosphorylate the KNL1 SLiMs). In contrast, PP1-KNL1 is redundant because even when it is removed, PP2A-B56 is still preserved on kinetochores

to antagonize Aurora B (Figure 1H). Therefore, the different forms of SLiM interaction explain why chromosomal alignment is primarily controlled by distinct phosphatase complexes at kinetochores (Figure 1A).

To determine the reason for the differential effect on the SAC, we focused on the crucial observation that PP1-KNL1 inhibition prevents SAC silencing even though PP2A-B56 remains bound to BUBR1 at kinetochores (Figures 1D and 1H). We hypothesized that the phospho-dependence of this BUBR1 interaction restricts PP2A-B56 from efficiently silencing the SAC, which is supported by the observation that PP1 can silence the SAC when recruited to BUBR1 in a manner that is independent of phosphorylation (BUBR1$^{\Delta CT\text{-}PP1:KNL1}$; Figure 3E). In agreement with this hypothesis, a similar effect is also observed when B56 is fused directly to BUBR1 (BUBR1$^{\Delta CT}$-B56γ; Figure 4D), which demonstrates that PP1 and PP2A-B56 can both silence the SAC efficiently when tethered directly to BUBR1, even in KNL1$^{\Delta PP1}$ cells. These fusions eliminate the dependence on phosphorylation for phosphatase recruitment, but, in addition, they also change the relative orientation of the phosphatases at BUBR1. This could, in principle, provide the additional flexibility needed for access to key substrates that might otherwise be inaccessible when B56 is bound to the LxxIxE motif. Increased flexibility is unlikely to explain why PP2A-B56 cannot silence the SAC, however, given that insertion of a flexible linker immediately before the PP2A binding motif in BUBR1 does not affect MELT dephosphorylation or SAC silencing in either the presence or absence of PP1-KNL1 (Figure S4). Nevertheless, to test directly whether lack of phospho-dependence was the reason for enhanced SAC silencing, we mutated the PP2A binding sequence in BUBR1 to an LxxIxE sequence that binds to B56 in the same manner and with similar affinities but, crucially, does not depend on phosphorylation (BUBR1$^{deP\text{-}PP2A}$, which uses an LPTIHE sequence; Kruse et al., 2018). Figure 4E shows that BUBR1$^{deP\text{-}PP2A}$ cells were now able to silence the SAC in the absence of PP1-KNL1, demonstrating that PP2A-B56 is restricted from silencing the SAC because of a phospho-dependent interaction with BUBR1. There is still an additional contribution of PP1-KNL1 in this situation (compare BUBR1$^{deP\text{-}PP2A}$ in KNL1^{WT} and KNL1$^{\Delta PP1}$ cells), which likely indicates that both phosphatases collaborate to shut down the SAC. This is predicted given that both phosphatases are indistinguishable in our assays when they are coupled in either position, independent of phosphorylation (Figures 2B–2E).

In summary, although PP1 and PP2A-B56 are indistinguishable in our assays when their positions are switched (Figure 2), they can still produce distinct effects because they couple to phosphorylation inputs in opposite ways (Figure 4). Therefore, we next sought to address whether this alone was sufficient to explain their phenotypic differences at kinetochores. To this end, we developed a mathematical model of the network outlined in Figure 1I. A crucial aspect of this model, which is displayed schematically in Figure 5A, is that both phosphatases dephosphorylate the same substrates (KNL1-pMELT, KNL1-pRVSF, BUBR1-pLSPI, and pNDC80) with identical kinetics when docked to their native SLiMs on KNL1. This binding occurs directly for PP1 (via dephospho-KNL1-RVSF) or indirectly for PP2A (via phospho-KNL1-MELT and phospho-BUBR1-LSPI). The kinases that phosphorylate these docking motifs (MPS1, Aurora B, and CDK1) are given a fixed activity that is not regulated by the phosphatases or any other aspect of the model. Therefore, any difference between the two phosphatases in the model is due to their inverse phospho-dependencies, as suggested by all results presented so far.

Modeling to Show How Identical Phosphatases with Opposite Phospho-dependencies Can Produce Distinct Phenotypic Behaviors

We first asked whether the model could reproduce the SAC data from Figures 1C–1F. In our simulations, KNL1-MELT is dephosphorylated rapidly upon MPS1 inhibition, and this is dependent on the presence of both PP1 and PP2A. Combined Aurora B inhibition speeds up the rate of MELT dephosphorylation and specifically rescues the effects of PP2A loss (Figures 5B and 5C). Therefore, the model can reproduce the core data, which crucially, also includes one key unexplained aspect of our results: PP2A-B56 is unable to silence the SAC in the absence of PP1. We were able to explore this further in the model to demonstrate that negative feedback loops downstream of PP2A prevent this phosphatase from efficiently silencing the SAC. Negative feedback occurs at multiple levels because PP2A dephosphorylates both BUBR1 and KNL1 to effectively remove and inhibit its own recruiting SLiM (this occurs directly and indirectly via PP1; Figure 5D). We had already determined how much of an effect these feedback loops have on PP2A-B56 localization by quantifying kinetochore B56γ levels following expression of a WT or inactive B56 mutant (B56γ^{CD}; Figure S5) and then including this difference in the model. Now, by selectively removing the loops, we can measure their effects on the output. This demonstrates that preventing the feedback onto phospho-BUBR1 reduces phospho-MELT levels (Figure 5E), which is consistent with the fact that PP2A can silence the SAC effectively in BUBR1-B56 or BUBR1$^{deP\text{-}PP2A}$ cells (Figures 4D and 4E). However, a stronger effect on MELT dephosphorylation is observed when all negative feedback loops are abolished by recruiting a phosphatase that is independent of either phospho-BUBR1 or phospho-KNL1 (Figure 5E). In this case, there is also a significantly increased sensitivity to changes in MPS1 levels. This is effectively the situation that is achieved when Aurora B is inhibited and PP1-KNL1 recruitment to kinetochores is unconstrained, as can be seen in the WT situation with Aurora B inhibition (Figure 5E).

Therefore, the model illustrates how negative feedback downstream of PP2A could allow the SAC to remain robust to variations in MPS1 activity (i.e., KNL1-pMELT levels remain high when MPS1 levels decrease; Figure 5E) by limiting the ability of this phosphatase to dephosphorylate the MELTs on its own. When Aurora B activity falls at kinetochores, then PP1 recruitment is elevated, and the SAC can be efficiently silenced without the effects of negative feedback restricting phosphatase levels. In fact, this transition is aided by positive feedback instead because PP1 dephosphorylates the KNL1-RVSF motif to enhance its own recruitment (Figure 5D). Using parameters identical to the SAC simulation (STAR Methods), the model also simulates the cross-regulation that is illustrated in Figure 4A. In the absence of PP2A, PP1 levels are dramatically reduced,

OPEN
ACCESS
CellPress

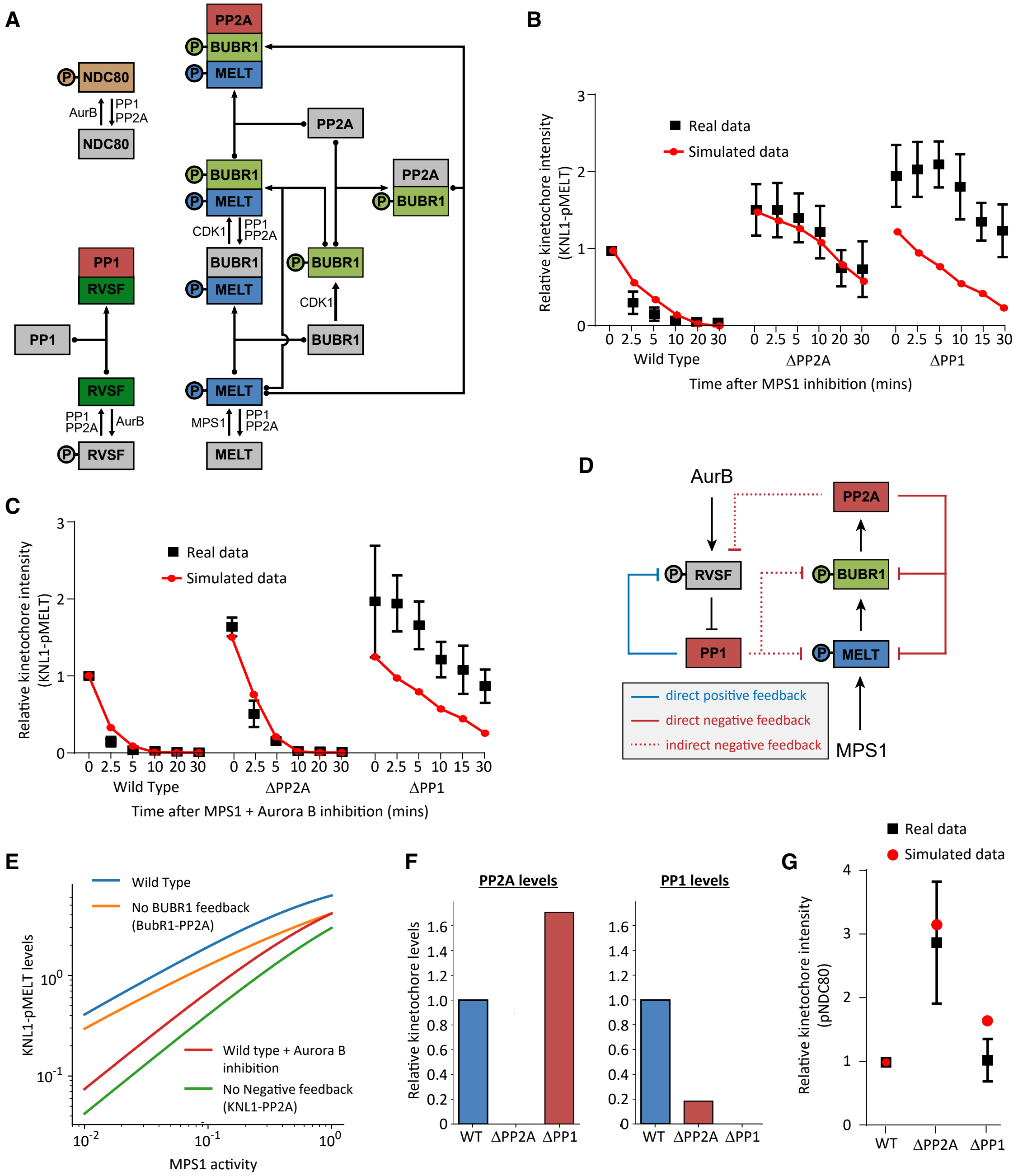

Figure 5. Mathematical Model Showing How Identical Phosphatases with Opposite Phospho-dependencies Can Produce Distinct Phenotypic Behaviors

(A) Full wiring diagram underlying the mathematical model that assumes identical activities of PP1 and PP2A toward all kinetochore substrates. Their only differences are their opposite modes of phospho-regulation. Arrows with dotted ends represent reversible binding/dissociation reactions. Regular arrows represent phosphorylation/dephosphorylation reactions catalyzed by the kinases/phosphatases, as indicated.

(B and C) Comparison of the simulated output with the real data from Figures 1C–1F. The graphs show KNL1-MELT dephosphorylation after MPS1 inhibition (B) and MPS1 + Aurora B inhibition (C).

(D) Schematic illustrating the various positive and negative feedback loops that affect phosphatase recruitment. Note that both phosphatases act on all phosphorylation sites.

(E) Simulated steady-state levels of KNL1-pMELT as a function of MPS1 for different conditions to remove negative feedback or inhibit Aurora B. Sensitivity/robustness can be compared directly because it corresponds to the slope of the curve on the log-scale graph.

(F) Simulated kinetochore levels of PP1 or PP2A after removal of either phosphatase. The simulated output confirms the predictions for cross-regulation in Figure 4A.

(G) Comparison of simulated pNDC80 steady-state values during a SAC arrest (i.e., MPS1 and Aurora B active) with the experimental data from Figure 1B.

whereas in the absence of PP1, PP2A levels are increased (Figure 5F). This is consistent with the data from Figure 1H and leads to a differential effect on steady-state pNDC80 levels (Figure 5G). This is also consistent with our observed differential effects on kinetochore-microtubule attachments and NDC80 phosphorylation (Figures 1A and 1B).

The simulation therefore illustrates how identical phosphatases can produce differential phenotypic behaviors by using opposite modes of phospho-regulation. Considering that we observed no other differences between PP1 and PP2A-B56 in any of our assays, this implies that kinetochores have evolved to interact with these phosphatases primarily because of their inverse phospho-dependencies. This has important implications for signaling in general because it is likely that many other pathways have also selected for these key defining features.

Phospho-regulation Is a Common Feature of RVxF and LxxIxE SLiMs

To analyze this further, we curated a list of validated and predicted RVxF and LxxIxE motifs that are present in almost 700 unique proteins (Table S1). Motif analysis demonstrates that serines and threonines are statistically enriched at positions within each motif where phosphorylation is known to inhibit (RVxF) or enhance (LxxIxE) phosphatase interaction (Figures 6A and 6B; Kim et al., 2003; Nasa et al., 2018; Hertz et al., 2016; Wang et al., 2016a, 2016b; Kumar et al., 2016). Furthermore, up to 25% of the validated motifs are known to be phosphorylated *in vivo*, and 50% of the RVxF and 100% of the LxxIxE motifs contain phosphorylatable residues at the key positions (Figures 6C–6E), which is a statistically significant enrichment (see amino acid matrices in Table S1). It should be noted that phosphorylation of residues outside of the core RVxF region can also inhibit PP1 binding (Kumar et al., 2016; Qian et al., 2015; Vagnarelli et al., 2011). Furthermore, the negatively charged surface that surrounds the RVxF pocket on PP1 (Figure 6F) could potentially mediate many other electrostatic interactions that are inhibitable by phosphorylation. Therefore, although only half of the core RVxF motifs contain phosphorylatable residues, the percentage that are phospho-regulatable is probably much higher. In contrast to PP1, the interaction between PP2A-B56 and LxxIxE motifs can be enhanced by phosphorylation inside of and immediately after the core motif. This is because the charged phosphate residues in the P2 and P7–P9 positions can make key electrostatic interactions with basic residues in a groove on B56 (Hertz et al., 2016; Wang et al., 2016a, 2016b; Figure 6G). Therefore, the binding pockets on PP1 and B56 appear to have evolved to respond to phosphorylated SLiMs in opposite ways, and numerous pathways have likely taken advantage of these unique properties to enable localized phosphatase activity to be modulated by different kinase inputs. This study therefore provides a paradigm that could explain how two of the main phosphatase families in eukaryotic cells have evolved to perform very important but also very distinct signaling roles.

DISCUSSION

The integration of kinase and phosphatase signals is a critical aspect of signal transduction (Gelens et al., 2018). At the kinetochore, numerous kinase and phosphatase signals converge at the KMN network to regulate two key mitotic processes (Saurin, 2018). We show here that the two distinct phosphatases, in this case, are used for their ability to positively or negatively respond to kinase inputs. Although the kinases themselves clearly play an important role in determining phosphatase function, as will be discussed further below, it is important to point out that the differential effects illustrated here are primarily caused by cross-regulation and feedback between phosphatases.

The cross-regulation occurs because the phosphatases are embedded in the same network and can therefore dephosphorylate the respective SLiMs to produce opposing effects on each other's recruitment (Figures 4A and 5F). In particular, PP2A can dephosphorylate the SILK and RVSF SLiMs to enhance PP1-KNL1 levels and exert control over net phosphatase activity at the KMN network. This control is relinquished upon loss of Aurora B activity because these SLiMs are no longer phosphorylated, which ensures that PP1-KNL1 can then be recruited independently of PP2A-B56. This is likely to be important to allow SAC silencing and microtubule stability to be maintained when PP2A-B56 is removed from kinetochores under tension.

In addition to cross-regulation, their unique phospho-dependencies also elicit different forms of feedback regulation. There are many feedback loops to consider (Figure 5D), but the underlying theme is that phosphatase activity helps to enhance PP1 and repress PP2A. As a consequence, PP2A is subjected to a variety of negative feedback loops, which, as our modeling suggests, could be one reason that this phosphatase cannot efficiently dephosphorylate the MELT motifs on its own following MPS1 inhibition. This contrasts with PP1, which engages in positive feedback and can dephosphorylate the MELT motifs in a more responsive manner (Figure 5E). Aurora B activity safeguards the transition from PP2A to PP1 at kinetochores, which illustrates why it plays a key role in determining whether the SAC is robust or responsive to declining MPS1 activity. This has two important implications. (1) Tension is ultimately required to inhibit Aurora B and allow the SAC to be silenced efficiently. This may explain why the SAC remains active on mono-orientated attachments and why it still takes hours to exit mitosis when stable attachments are formed that cannot generate tension (Tauchman et al., 2015; Etemad and Kops, 2016). (2) Aurora B is a potentially dangerous node in the network that could be hijacked by cancer cells to weaken the SAC and generate hyper-stable kinetochore-microtubule attachments (Cordeiro et al., 2018). These two effects can collaborate to generate high levels of chromosomal instability; therefore, it will be important in the future to determine whether Aurora B activity is commonly deregulated in cancer cells.

Another important implication of this work is that kinetochore-microtubule attachments and the SAC are principally regulated by different phosphatase complexes. We show that, despite their lack of specificity, PP2A-B56 is essential to stabilize microtubule attachments, but PP1-KNL1 is then ultimately required to help shut down the SAC. A similar separation of function was recently demonstrated in yeast, but, in this case, between different PP1 complexes (Roy et al., 2018). The general principle is the same in both cases, however, because sequential regulation by different phosphatases is predicted to guard against

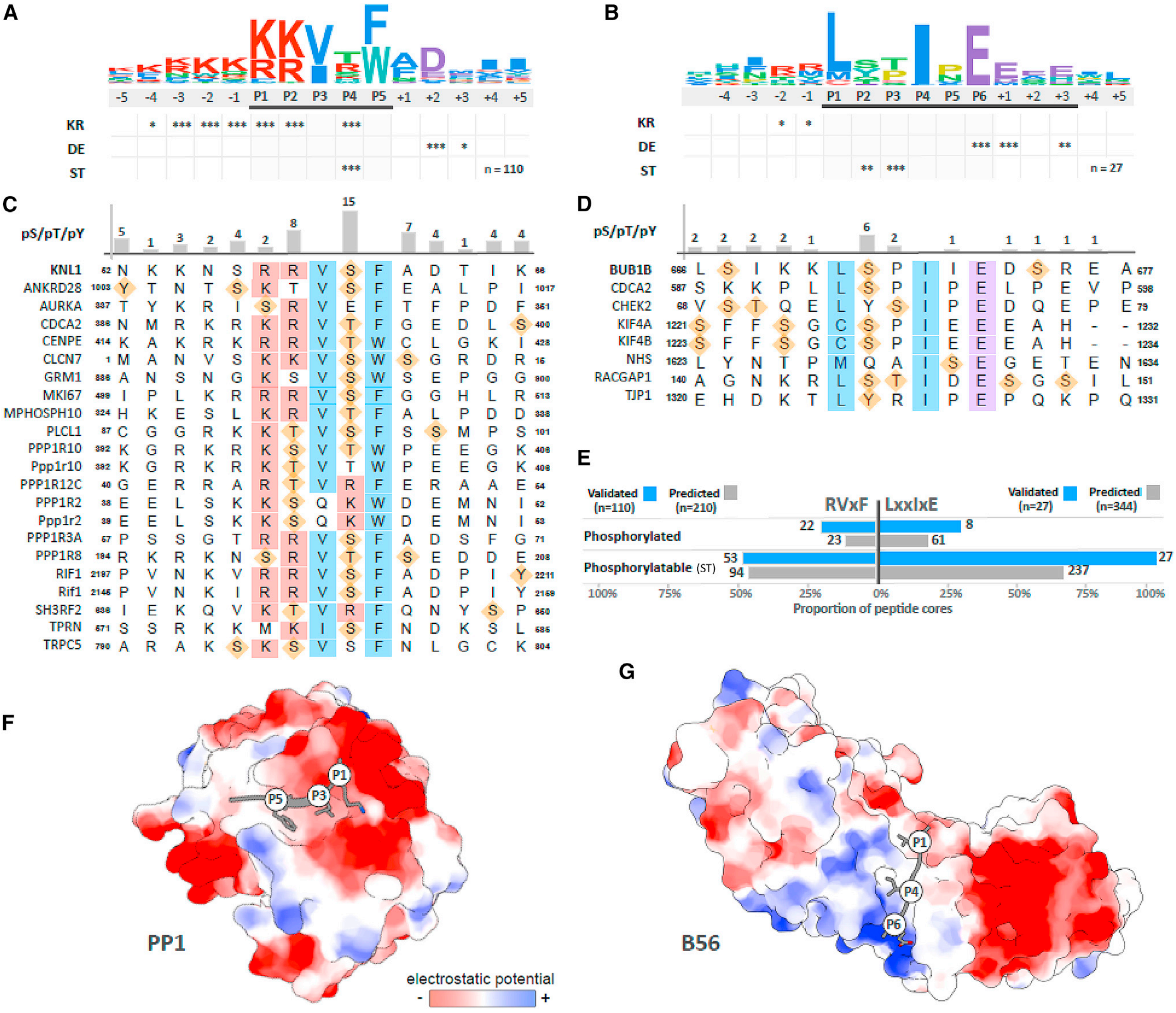

Figure 6. Analysis of the Specificity Determinants and Phosphorylation Sites in PP1 RVxF and PP2A-B56 LxxIxE Motifs

(A) A log10 relative binomial sequence logo based on 110 validated PP1-binding RVxF motifs. Asterisks denote the significance of the amino acid enrichment of basic (KR), acidic (DE), and phosphorylatable by Ser/Thr kinases (ST) (*p < 0.01, **p < 0.001, *** p < 0.0001). The logo is colored using ClustalW coloring.

(B) As in (A), built using 27 validated PP2A-B56-binding LxxIxE motifs.

(C) Sequence of the 22 RVxF motifs with experimentally validated phosphorylation sites within the region of the motif (the region defined by the black line under the logo in A). Consensus positions are indicated by boxes colored according to the ClustalW coloring scheme. Phosphorylated sites are indicated with orange diamonds.

(D) As in C, but for the 8 LxxIxE motifs with experimentally validated phosphorylation sites.

(E) Summary of the LxxIxE and RVxF motifs phosphorylated (experimentally validated) and phosphorylatable (ST) within the motif regions (defined by the black lines under the logos in A and B). Data are shown for validated motifs (blue bars) and a set of high-confidence predicted motifs (gray bars) created using the PSSMSearch software (Krystkowiak et al., 2018) by using the validated motifs as input and filtering as described in Hertz et al. (2016).

(F) Structure of the RVxF motif of Rb bound to PP1 showing the key side chains of the motif that interact with the binding pocket (PDB: 3N5U; Hirschi et al., 2010).

(G) Structure of the LxxIxE motif of BUBR1 bound to B56, showing the key side chains of the motif that interact with the binding pocket (PDB: 5JJA; Wang et al., 2016a). Structures are rendered using Coulombic surface coloring in the Chimera package to show the surface charge around the motif binding pockets.

inappropriate SAC silencing when microtubule attachments are not correct.

The work presented here implies that kinetochores have evolved to interact with PP1 and PP2A-B56 mainly because of their opposite phospho-dependencies. However, it is important to clarify that, although these phosphatases were fully interchangeable in all our experiments, this does not exclude the possibility that important differences exist that were simply not detected in our assays. The relative activities of each phosphatase toward key phosphorylation sites on KNL1 seem to be

identical (Figures 2B, 2D, and 4B), but there are probably other important substrates that remain to be measured. Furthermore, there are other established regulatory mechanisms that do not involve the SLiMs but that could be needed for some aspects of kinetochore regulation (Grallert et al., 2015; Porter et al., 2013). The notion that important differences could simply have been missed by our assays is illustrated nicely by our experiments on the RVSF motif in KNL1. The N-terminal position of this SLiM is not important for SAC silencing in the absence of microtubules, but the strict conservation at this position throughout evolution indicates that there is an essential requirement that still remains to be discovered (Figures 3C, 3D, and S3). Interestingly, the microtubule binding site on KNL1 has been shown recently to overlap the PP1-binding SLiMs (Bajaj et al., 2018). Therefore, we speculate that microtubules might need to compete with PP1 at the N terminus. For example, they may need to elongate the KNL1 structure and disrupt PP1 binding at the same time. It will be interesting to test how this competition could affect Aurora B regulation, error correction, and tension sensing.

Although other properties of PP1 and PP2A could be important in some contexts, their inverse phospho-dependencies are clearly the defining features with regard to the SAC and kinetochore-microtubule attachments. This explains why the relevant phosphorylation sites are so well-conserved within each kinetochore SLiM (Figure 3B; Suijkerbuijk et al., 2012). What then, is the relevance of the particular kinase inputs needed to phosphorylate these SLiMs? As discussed previously, Aurora B may regulate the PP1 SLiMs to allow the SAC phosphatase to respond to tension. We speculate that PLK1 and CDK1 may similarly regulate the PP2A-B56 SLiM to allow the kinetochore-microtubule phosphatase to respond to microtubule attachment. This is predicted given that both kinases are recruited to kinetochores in an attachment-sensitive manner (Allan et al., 2019; Jackman et al., 2019; Alfonso-Pérez et al., 2019; Lénárt et al., 2007; Liu et al., 2012). In fact, both are also recruited to the KMN network in a phosphorylation-dependent manner: Cyclin B/CDK1 interacts with Mad1, a phospho-dependent interactor of BUB1, and CDK1 can phosphorylate BUB1 to recruit PLK1 (Allan et al., 2019; Jackman et al., 2019; Alfonso-Pérez et al., 2019; Saurin, 2018). Interestingly, the key Mad1-BUB1 interaction has also been shown to be negatively regulated by kinetochore PP2A-B56 (Qian et al., 2017). Therefore, PP2A may also counteract CDK1 and PLK1 activity to create yet more negative feedback to restrict its activity. This would serve to restrict PP2A from silencing the SAC even more, which could allow the current mathematical model to better approximate the real data. It should be stressed that this model is just a basic framework to explore how inverse phospho-dependencies could affect phosphatase behaviors. The output of the modeling should be viewed as a proof of principle rather than proof of behavior. In the future, details of how the various kinase inputs respond to phosphatase activity should be layered into this model to provide a more complete picture of signal integration at the KMN network.

Finally, if kinetochores have selected to interact with PP1 and PP2A-B56 because of their inverse phospho-dependencies, then many other pathways have likely exploited the same features. This would explain the prevalence of phosphorylation sites within validated and predicted RVxF and LxxIxE SLiMs (Figures 6C–6E; Table S1). The invariant SLiM residues place some constraints on the type of kinase inputs that are tolerated within the motifs themselves (Figure S6), but phosphorylation outside of these regions can also regulate phosphatase binding (Kumar et al., 2016; Qian et al., 2015; Vagnarelli et al., 2011). Furthermore, PP1 uses co-operative interaction with other SLiMs, and some of these, such as the SILK motif in KNL1, are also phospho-inhibitable (Liu et al., 2010). Therefore, these additional interactions could allow a wide range of kinase inputs to converge and fine-tune PP1 binding strength. This is analogous to the contribution of both CDK1 and PLK1 to PP2A-B56 binding on BUBR1 (Huang et al., 2008; Elowe et al., 2007; Kruse et al., 2013; Suijkerbuijk et al., 2012; Wang et al., 2016a, 2016b). It will be important in the future to fully characterize all possible SLiM interactors for both PP1 and PP2A-B56 and to determine how these can be regulated by different kinases. As pointed out recently, kinases and phosphatases work together in many different ways to generate the right type of signal response (Gelens et al., 2018; Gelens and Saurin, 2018). Therefore, the ability of different phosphatases to positively or negatively couple to phosphorylation inputs represents a fundamental but still poorly understood aspect of signal integration.

STAR★METHODS

Detailed methods are provided in the online version of this paper and include the following:

- KEY RESOURCES TABLE
- LEAD CONTACT AND MATERIALS AVAILABILITY
- EXPERIMENTAL MODEL AND SUBJECT DETAILS
- METHOD DETAILS
  - Plasmids and cloning
  - Gene expression
  - Gene knockdown
  - Immunofluorescence
  - Chromosome alignment assays
  - Time-lapse analyses
  - Antibodies
  - Mathematical modeling approach, terminology and assumptions
  - Modeling equations
  - Free amounts and total amounts for the modeling
  - Implementation of mutants and parameter optimization
  - Computational methods
  - Motif analysis
- QUANTIFICATION AND STATISTICAL ANALYSIS
- DATA AND CODE AVAILABILITY

SUPPLEMENTAL INFORMATION

Supplemental Information can be found online at https://doi.org/10.1016/j.celrep.2019.07.067.

ACKNOWLEDGMENTS

M.H.C., R.J.S., and A.T.S. are funded by Cancer Research UK C47320/A21229 and C10988/A22566 (to A.T.S.). G.V. was funded by Tenovus

Scotland T14-19 (to A.T.S.). A.C. is funded by a grant from the Italian Association for Cancer Research (IG 21556). N.E.D. was supported by an Science Foundation Ireland (SFI) starting investigator research grant (13/SIRG/2193). We thank the staff at the Dundee Imaging Facility, the Flow Cytometry and Cell Sorting Facility, and the Genetic Core Services Unit. We also thank Stephen Taylor for providing the HeLa Flp-in cell line and Geert Kops, Peter Parker, and Iain Cheeseman for antibodies.

AUTHOR CONTRIBUTIONS

A.T.S., R.J.S., and M.H.C. conceived the study, designed the experiments, and interpreted the data. R.J.S. and M.H.C. performed the majority of these experiments with contributions from G.V. F.G. and A.C. performed the modeling for Figure 5. N.E.D. performed the SLiM analysis for Figure 6 and Table S1. A.T.S. wrote the manuscript with input from all authors.

DECLARATION OF INTERESTS

The authors declare no competing interests.

Received: March 1, 2019
Revised: June 18, 2019
Accepted: July 18, 2019
Published: August 20, 2019

SUPPORTING CITATIONS

The following references appear in the Supplemental Information: Alexander et al. (2011); Tromer et al. (2015).

REFERENCES

Alexander, J., Lim, D., Joughin, B.A., Hegemann, B., Hutchins, J.R., Ehrenberger, T., Ivins, F., Sessa, F., Hudecz, O., Nigg, E.A., et al. (2011). Spatial exclusivity combined with positive and negative selection of phosphorylation motifs is the basis for context-dependent mitotic signaling. Sci. Signal. *4*, ra42.

Alfonso-Pérez, T., Hayward, D., Holder, J., Gruneberg, U., and Barr, F.A. (2019). MAD1-dependent recruitment of CDK1-CCNB1 to kinetochores promotes spindle checkpoint signaling. J. Cell Biol. *218*, 1108–1117.

Allan, L.A., Reis, M., Liu, Y., Huis in 't Veld, P., Kops, G.J., Musacchio, A., and Saurin, A.T. (2019). Cyclin B1 scaffolds MAD1 at the corona to activate the spindle assembly checkpoint. bioRxiv. https://doi.org/10.1101/726224.

Altschul, S.F., Madden, T.L., Schäffer, A.A., Zhang, J., Zhang, Z., Miller, W., and Lipman, D.J. (1997). Gapped BLAST and PSI-BLAST: a new generation of protein database search programs. Nucleic Acids Res. *25*, 3389–3402.

Bajaj, R., Bollen, M., Peti, W., and Page, R. (2018). KNL1 Binding to PP1 and Microtubules Is Mutually Exclusive. Structure *26*, 1327–1336.e4.

Brautigan, D.L., and Shenolikar, S. (2018). Protein Serine/Threonine Phosphatases: Keys to Unlocking Regulators and Substrates. Annu. Rev. Biochem. *87*, 921–964.

Cordeiro, M.H., Smith, R.J., and Saurin, A.T. (2018). A fine balancing act: A delicate kinase-phosphatase equilibrium that protects against chromosomal instability and cancer. Int. J. Biochem. Cell Biol. *96*, 148–156.

Edelstein, A., Amodaj, N., Hoover, K., Vale, R., and Stuurman, N. (2010). Computer control of microscopes using μManager. Current Protocols in Molecular Biology *92*, 14–20.

Elowe, S., Hümmer, S., Uldschmid, A., Li, X., and Nigg, E.A. (2007). Tension-sensitive Plk1 phosphorylation on BubR1 regulates the stability of kinetochore microtubule interactions. Genes Dev. *21*, 2205–2219.

Espert, A., Uluocak, P., Bastos, R.N., Mangat, D., Graab, P., and Gruneberg, U. (2014). PP2A-B56 opposes Mps1 phosphorylation of Knl1 and thereby promotes spindle assembly checkpoint silencing. J. Cell Biol. *206*, 833–842.

Espeut, J., Cheerambathur, D.K., Krenning, L., Oegema, K., and Desai, A. (2012). Microtubule binding by KNL-1 contributes to spindle checkpoint silencing at the kinetochore. J. Cell Biol. *196*, 469–482.

Etemad, B., and Kops, G.J. (2016). Attachment issues: kinetochore transformations and spindle checkpoint silencing. Curr. Opin. Cell Biol. *39*, 101–108.

Ferrell, J.E., Jr., and Ha, S.H. (2014). Ultrasensitivity part I: Michaelian responses and zero-order ultrasensitivity. Trends Biochem. Sci. *39*, 496–503.

Foley, E.A., Maldonado, M., and Kapoor, T.M. (2011). Formation of stable attachments between kinetochores and microtubules depends on the B56-PP2A phosphatase. Nat. Cell Biol. *13*, 1265–1271.

Gelens, L., and Saurin, A.T. (2018). Exploring the Function of Dynamic Phosphorylation-Dephosphorylation Cycles. Dev. Cell *44*, 659–663.

Gelens, L., Qian, J., Bollen, M., and Saurin, A.T. (2018). The Importance of Kinase-Phosphatase Integration: Lessons from Mitosis. Trends Cell Biol. *28*, 6–21.

Gharbi-Ayachi, A., Labbé, J.C., Burgess, A., Vigneron, S., Strub, J.M., Brioudes, E., Van-Dorsselaer, A., Castro, A., and Lorca, T. (2010). The substrate of Greatwall kinase, Arpp19, controls mitosis by inhibiting protein phosphatase 2A. Science *330*, 1673–1677.

Grallert, A., Boke, E., Hagting, A., Hodgson, B., Connolly, Y., Griffiths, J.R., Smith, D.L., Pines, J., and Hagan, I.M. (2015). A PP1-PP2A phosphatase relay controls mitotic progression. Nature *517*, 94–98.

Heroes, E., Lesage, B., Görnemann, J., Beullens, M., Van Meervelt, L., and Bollen, M. (2013). The PP1 binding code: a molecular-lego strategy that governs specificity. FEBS J. *280*, 584–595.

Hertz, E.P.T., Kruse, T., Davey, N.E., López-Méndez, B., Sigurðsson, J.O., Montoya, G., Olsen, J.V., and Nilsson, J. (2016). A Conserved Motif Provides Binding Specificity to the PP2A-B56 Phosphatase. Mol. Cell *63*, 686–695.

Hirschi, A., Cecchini, M., Steinhardt, R.C., Schamber, M.R., Dick, F.A., and Rubin, S.M. (2010). An overlapping kinase and phosphatase docking site regulates activity of the retinoblastoma protein. Nat. Struct. Mol. Biol. *17*, 1051–1057.

Huang, H., Hittle, J., Zappacosta, F., Annan, R.S., Hershko, A., and Yen, T.J. (2008). Phosphorylation sites in BubR1 that regulate kinetochore attachment, tension, and mitotic exit. J. Cell Biol. *183*, 667–680.

Ingebritsen, T.S., and Cohen, P. (1983). The protein phosphatases involved in cellular regulation. 1. Classification and substrate specificities. Eur. J. Biochem. *132*, 255–261.

Jackman, M., Marcozzi, C., Pardo, M., Yu, L., Tyson, A.L., Choudhary, J.S., and Pines, J. (2019). Cyclin B1-Cdk1 binding to MAD1 links nuclear pore disassembly to chromosomal stability. bioRxiv. https://doi.org/10.1101/701474.

Kauko, O., Imanishi, S.Y., Kulesskiy, E., Laajala, T.D., Yetukuri, L., Laine, A., Jumppanen, M., Haapaniemi, P., Ruan, L., Yadav, B., et al. (2018). Rules for PP2A-controlled phosphosignalling and drug responses. bioRxiv. https://doi.org/10.1101/271841.

Kim, Y.M., Watanabe, T., Allen, P.B., Kim, Y.M., Lee, S.J., Greengard, P., Nairn, A.C., and Kwon, Y.G. (2003). PNUTS, a protein phosphatase 1 (PP1) nuclear targeting subunit. Characterization of its PP1- and RNA-binding domains and regulation by phosphorylation. J. Biol. Chem. *278*, 13819–13828.

Krenn, V., and Musacchio, A. (2015). The Aurora B Kinase in Chromosome Bi-Orientation and Spindle Checkpoint Signaling. Front. Oncol. *5*, 225.

Kruse, T., Zhang, G., Larsen, M.S., Lischetti, T., Streicher, W., Kragh Nielsen, T., Bjørn, S.P., and Nilsson, J. (2013). Direct binding between BubR1 and B56-PP2A phosphatase complexes regulate mitotic progression. J. Cell Sci. *126*, 1086–1092.

Kruse, T., Biedenkopf, N., Hertz, E.P.T., Dietzel, E., Stalmann, G., Lopez-Mendez, B., Davey, N.E., Nilsson, J., and Becker, S. (2018). The Ebola Virus Nucleoprotein Recruits the Host PP2A-B56 Phosphatase to Activate Transcriptional Support Activity of VP30. Mol. Cell *69*, 136–145.e6.

Krystkowiak, I., Manguy, J., and Davey, N.E. (2018). PSSMSearch: a server for modeling, visualization, proteome-wide discovery and annotation of protein motif specificity determinants. Nucleic Acids Res. *46* (*W1*), W235–W241.

Kumar, G.S., Gokhan, E., De Munter, S., Bollen, M., Vagnarelli, P., Peti, W., and Page, R. (2016). The Ki-67 and RepoMan mitotic phosphatases assemble via an identical, yet novel mechanism. eLife *5*, e16539.

Lénárt, P., Petronczki, M., Steegmaier, M., Di Fiore, B., Lipp, J.J., Hoffmann, M., Rettig, W.J., Kraut, N., and Peters, J.M. (2007). The small-molecule inhibitor BI 2536 reveals novel insights into mitotic roles of polo-like kinase 1. Curr. Biol. *17*, 304–315.

Liu, D., Vleugel, M., Backer, C.B., Hori, T., Fukagawa, T., Cheeseman, I.M., and Lampson, M.A. (2010). Regulated targeting of protein phosphatase 1 to the outer kinetochore by KNL1 opposes Aurora B kinase. J. Cell Biol. *188*, 809–820.

Liu, D., Davydenko, O., and Lampson, M.A. (2012). Polo-like kinase-1 regulates kinetochore-microtubule dynamics and spindle checkpoint silencing. J. Cell Biol. *198*, 491–499.

London, N., Ceto, S., Ranish, J.A., and Biggins, S. (2012). Phosphoregulation of Spc105 by Mps1 and PP1 regulates Bub1 localization to kinetochores. Curr. Biol. *22*, 900–906.

Maldonado, M., and Kapoor, T.M. (2011). Constitutive Mad1 targeting to kinetochores uncouples checkpoint signalling from chromosome biorientation. Nat. Cell Biol. *13*, 475–482.

Meadows, J.C., Shepperd, L.A., Vanoosthuyse, V., Lancaster, T.C., Sochaj, A.M., Buttrick, G.J., Hardwick, K.G., and Millar, J.B. (2011). Spindle checkpoint silencing requires association of PP1 to both Spc7 and kinesin-8 motors. Dev. Cell *20*, 739–750.

Mochida, S., Maslen, S.L., Skehel, M., and Hunt, T. (2010). Greatwall phosphorylates an inhibitor of protein phosphatase 2A that is essential for mitosis. Science *330*, 1670–1673.

Myers, C.R., Gutenkunst, R.N., and Sethna, J.P. (2007). Python unleashed on systems biology. Comput. Sci. Eng. *9*, 34–37.

Nasa, I., Rusin, S.F., Kettenbach, A.N., and Moorhead, G.B. (2018). Aurora B opposes PP1 function in mitosis by phosphorylating the conserved PP1-binding RVxF motif in PP1 regulatory proteins. Sci. Signal. *11*, eaai8669.

Nijenhuis, W., Vallardi, G., Teixeira, A., Kops, G.J., and Saurin, A.T. (2014). Negative feedback at kinetochores underlies a responsive spindle checkpoint signal. Nat. Cell Biol. *16*, 1257–1264.

Overlack, K., Primorac, I., Vleugel, M., Krenn, V., Maffini, S., Hoffmann, I., Kops, G.J., and Musacchio, A. (2015). A molecular basis for the differential roles of Bub1 and BubR1 in the spindle assembly checkpoint. eLife *4*, e05269.

Porter, I.M., Schleicher, K., Porter, M., and Swedlow, J.R. (2013). Bod1 regulates protein phosphatase 2A at mitotic kinetochores. Nat. Commun. *4*, 2677.

Primorac, I., Weir, J.R., Chiroli, E., Gross, F., Hoffmann, I., van Gerwen, S., Ciliberto, A., and Musacchio, A. (2013). Bub3 reads phosphorylated MELT repeats to promote spindle assembly checkpoint signaling. eLife *2*, e01030.

Qian, J., Beullens, M., Huang, J., De Munter, S., Lesage, B., and Bollen, M. (2015). Cdk1 orders mitotic events through coordination of a chromosome-associated phosphatase switch. Nat. Commun. *6*, 10215.

Qian, J., Garcia-Gimeno, M.A., Beullens, M., Manzione, M.G., Van der Hoeven, G., Igual, J.C., Heredia, M., Sanz, P., Gelens, L., and Bollen, M. (2017). An Attachment-Independent Biochemical Timer of the Spindle Assembly Checkpoint. Mol. Cell *68*, 715–730.e5.

Rogers, S., McCloy, R., Watkins, D.N., and Burgess, A. (2016). Mechanisms regulating phosphatase specificity and the removal of individual phosphorylation sites during mitotic exit. BioEssays *38* (*Suppl 1*), S24–S32.

Rosenberg, J.S., Cross, F.R., and Funabiki, H. (2011). KNL1/Spc105 recruits PP1 to silence the spindle assembly checkpoint. Curr. Biol. *21*, 942–947.

Roy, B., Verma, V., Sim, J., Fontan, A., and Joglekar, A.P. (2018). Minimization of cross-talk between Spindle Assembly Checkpoint silencing and error correction. bioRxiv. https://doi.org/10.1101/459594.

Saurin, A.T. (2018). Kinase and Phosphatase Cross-Talk at the Kinetochore. Front. Cell Dev. Biol. *6*, 62.

Saurin, A.T., van der Waal, M.S., Medema, R.H., Lens, S.M., and Kops, G.J. (2011). Aurora B potentiates Mps1 activation to ensure rapid checkpoint establishment at the onset of mitosis. Nat. Commun. *2*, 316.

Schindelin, J., Arganda-Carreras, I., Frise, E., Kaynig, V., Longair, M., Pietzsch, T., Preibisch, S., Rueden, C., Saalfeld, S., Schmid, B., and Tinevez, J.Y. (2012). Fiji: an open-source platform for biological-image analysis. Nature Methods *9*, 676.

Shepperd, L.A., Meadows, J.C., Sochaj, A.M., Lancaster, T.C., Zou, J., Buttrick, G.J., Rappsilber, J., Hardwick, K.G., and Millar, J.B. (2012). Phosphodependent recruitment of Bub1 and Bub3 to Spc7/KNL1 by Mph1 kinase maintains the spindle checkpoint. Curr. Biol. *22*, 891–899.

Shrestha, R.L., Conti, D., Tamura, N., Braun, D., Ramalingam, R.A., Cieslinski, K., Ries, J., and Draviam, V.M. (2017). Aurora-B kinase pathway controls the lateral to end-on conversion of kinetochore-microtubule attachments in human cells. Nat. Commun. *8*, 150.

Suijkerbuijk, S.J., Vleugel, M., Teixeira, A., and Kops, G.J. (2012). Integration of kinase and phosphatase activities by BUBR1 ensures formation of stable kinetochore-microtubule attachments. Dev. Cell *23*, 745–755.

Tauchman, E.C., Boehm, F.J., and DeLuca, J.G. (2015). Stable kinetochore-microtubule attachment is sufficient to silence the spindle assembly checkpoint in human cells. Nat. Commun. *6*, 10036.

Tighe, A., Staples, O., and Taylor, S. (2008). Mps1 kinase activity restrains anaphase during an unperturbed mitosis and targets Mad2 to kinetochores. J. Cell Biol. *181*, 893–901.

Tromer, E., Snel, B., and Kops, G.J. (2015). Widespread Recurrent Patterns of Rapid Repeat Evolution in the Kinetochore Scaffold KNL1. Genome Biol. Evol. *7*, 2383–2393.

Vagnarelli, P., Ribeiro, S., Sennels, L., Sanchez-Pulido, L., de Lima Alves, F., Verheyen, T., Kelly, D.A., Ponting, C.P., Rappsilber, J., and Earnshaw, W.C. (2011). Repo-Man coordinates chromosomal reorganization with nuclear envelope reassembly during mitotic exit. Dev. Cell *21*, 328–342.

Vallardi, G., Allan, L.A., Crozier, L., and Saurin, A.T. (2019). Division of labour between PP2A-B56 isoforms at the centromere and kinetochore. eLife *8*, e42619.

Verbinnen, I., Ferreira, M., and Bollen, M. (2017). Biogenesis and activity regulation of protein phosphatase 1. Biochem. Soc. Trans. *45*, 89–99.

Vleugel, M., Tromer, E., Omerzu, M., Groenewold, V., Nijenhuis, W., Snel, B., and Kops, G.J. (2013). Arrayed BUB recruitment modules in the kinetochore scaffold KNL1 promote accurate chromosome segregation. J. Cell Biol. *203*, 943–955.

Wang, J., Wang, Z., Yu, T., Yang, H., Virshup, D.M., Kops, G.J., Lee, S.H., Zhou, W., Li, X., Xu, W., and Rao, Z. (2016a). Crystal structure of a PP2A B56-BubR1 complex and its implications for PP2A substrate recruitment and localization. Protein Cell *7*, 516–526.

Wang, X., Bajaj, R., Bollen, M., Peti, W., and Page, R. (2016b). Expanding the PP2A Interactome by Defining a B56-Specific SLiM. Structure *24*, 2174–2181.

Welburn, J.P.I., Vleugel, M., Liu, D., Yates, J.R., Lampson, M.A., Fukagawa, T., and Cheeseman, I.M. (2010). Aurora B phosphorylates spatially distinct targets to differentially regulate the kinetochore-microtubule interface. Mol. Cell *14*, 383–392.

Xu, P., Raetz, E.A., Kitagawa, M., Virshup, D.M., and Lee, S.H. (2013). BUBR1 recruits PP2A via the B56 family of targeting subunits to promote chromosome congression. Biol. Open *2*, 479–486.

Yamagishi, Y., Yang, C.H., Tanno, Y., and Watanabe, Y. (2012). MPS1/Mph1 phosphorylates the kinetochore protein KNL1/Spc7 to recruit SAC components. Nat. Cell Biol. *14*, 746–752.

Zhang, G., Lischetti, T., and Nilsson, J. (2014). A minimal number of MELT repeats supports all the functions of KNL1 in chromosome segregation. J. Cell Sci. *127*, 871–884.

STAR★METHODS

KEY RESOURCES TABLE

REAGENT or RESOURCE	SOURCE	IDENTIFIER
Antibodies		
Chicken anti- GFP	Abcam	Cat# ab13970, RRID: AB_300798
Mouse anti- GFP	Gift from P. Parker	clone 4E12/8
Rabbit anti- NDC80 pS55	GeneTex	Cat# GTX70017, RRID:AB_11162004
Guinea pig anti- Cenp C	Caltag + Medsystems	Cat# PD030, RRID: AB_10693556
Rabbit anti- Bub1	Bethyl	Cat# A300-373A, RRID: AB_2065943
Mouse anti- BUBR1	Millipore	Cat# 05-898, RRID: AB_417374
Rabbit anti- mCherry	Genetex	Cat# GTX128508, RRID: AB_2721247
Rabbit anti- pMELT-KNL1 (pT943 and pT1155)	Gift from G.Kops	Nijenhuis et al., 2014
Rabbit anti- pRVSF-KNL1 (pS60-KNL1)	Gift from I. Cheeseman	Welburn et al., 2010
Goat anti- Chicken Alexa Fluor 488	Thermo Fisher	Cat# A-11039, RRID:AB_2534096
Goat anti- Mouse Alexa Fluor 488	Thermo Fisher	Cat# A-11029, RRID:AB_2534088
Goat anti- Rabbit Alexa Fluor 568	Thermo Fisher	Cat# A-11036, RRID:AB_10563566
Goat anti- Mouse Alexa Fluor 568	Thermo Fisher	Cat# A-11031, RRID:AB_144696
Goat anti- Guinea pig Alexa Fluor 647	Thermo Fisher	Cat# A-21450, RRID:AB_2735091
Goat anti- Rabbit Alexa Fluor 647	Thermo Fisher	Cat# A-31573, RRID:AB_2536183
Chemicals, Peptides, and Recombinant Proteins		
Doxycycline	Sigma-Aldrich	Cat# D9891
Thymidine	Sigma-Aldrich	Cat# T1895
Nocodazole	Sigma-Aldrich	Cat# 487928
puromycin	Santa Cruz biotechnology	Cat# sc-108071A
hygromycin B	Santa Cruz biotechnology	Cat# sc-29067
MG132 (proteasome inhibitor)	Sigma-Aldrich	Cat# 474787
AZ-3146 (MPS1 inhibitor)	Sigma-Aldrich	Cat# SML1427
ZM-447439 (Aurora B inhibitor)	Cayman Chemicals	Cat# CAY13601
RO-3306 (CDK1 inhibitor)	Tocris	Cat# 4181
Penicillin/Streptomycin	ThermoFisher Scientific	Cat# 15070–063
Deposited Data		
KVxF PP1 docking motif repository	http://slim.ucd.ie/pp2a/index.php?page=instances	N/A
PP2A-B56 binding motif repository	http://slim.ucd.ie/motifs/pp1/index.php?page=instances	N/A
Experimental Models: Cell Lines		
Human: HeLa Flp-in Empty	Gift from S. Taylor	Tighe et al., 2008
HeLa Flp-In YFP-BUBR1^{WT}	Nijenhuis et al., 2014	N/A
HeLa Flp-In YFP-BUBR1$^{\Delta PP2A}$	Nijenhuis et al., 2014	N/A
HeLa Flp-In YFP-BUBR1 $^{\Delta CT}$	This study	N/A
HeLa Flp-In YFP-BUBR1^{Long}	This study	N/A
HeLa Flp-In YFP-BUBR1$^{deP\text{-}PP2A}$	This study	N/A
HeLa Flp-In YFP-KNL1^{WT}	This study	N/A
HeLa Flp-In YFP-KNL1$^{\Delta PP1}$	This study	N/A
HeLa Flp-In YFP-KNL1$^{PP1(2A)}$	This study	N/A
HeLa Flp-In YFP-KNL1 $^{\Delta NT}$	This study	N/A
HeLa Flp-In YFP-B56γ_1-KNL1$^{\Delta NT}$	This study	N/A

(Continued on next page)

Continued

REAGENT or RESOURCE	SOURCE	IDENTIFIER
HeLa Flp-In YFP-B56 γ_1^{CD}-KNL1$^{\Delta NT}$	This study	N/A
HeLa Flp-In YFP-KNL1 $^{\Delta NT\text{-}PP1mid}$	This study	N/A
HeLa Flp-In YFP-BUBR1$^{\Delta CT\text{-}PP1:KNL1}$	This study	N/A
HeLa Flp-In YFP-BUBR1$^{\Delta CT\text{-}KNL1}$	This study	N/A
HeLa Flp-In YFP-BUBR1$^{\Delta CT}$-B56γ_1	This study	N/A
HeLa Flp-In YFP-KNL1^{WT} + mCherry-B56γ_1	This study	N/A
HeLa Flp-In YFP-KNL1$^{\Delta PP1}$ + mCherry-B56γ_1	This study	N/A
HeLa Flp-In YFP-BUBR1^{WT} + mCherry-B56γ_1	This study	N/A
HeLa Flp-In YFP-BUBR1 $^{\Delta CT}$ + mCherry-B56γ_1	This study	N/A
HeLa Flp-In YFP-BUBR1 $^{\Delta CT\text{-}PP1:KNL1}$ + mCherry-B56γ_1	This study	N/A
HeLa Flp-In YFP-BUBR1 $^{\Delta CT\text{-}KNL1}$ + mCherry-B56γ_1	This study	N/A
HeLa Flp-In YFP-B56γ_1	Vallardi et al., 2019	N/A
HeLa Flp-In YFP-B56γ_1^{CD}	Vallardi et al., 2019	N/A
Oligonucleotides		
siRNA targeting sequence: BUBR1	Sigma-Aldrich	N/A
dsiRNA targeting sequence: KNL1	Integrated DNA technologies	N/A
Recombinant DNA		
pcDNA5-YFP-BUBR1^{WT}	Nijenhuis et al., 2014	N/A
pcDNA5-YFP-BUBR1$^{\Delta PP2A}$	Nijenhuis et al., 2014	N/A
pcDNA5-YFP-BUBR1 $^{\Delta CT}$	This study	N/A
pcDNA5-YFP-BUBR1^{Long}	This study	N/A
pcDNA5-YFP-BUBR1$^{deP\text{-}PP2A}$	This study	N/A
pcDNA5-YFP-KNL1^{WT}	This study	N/A
pcDNA5-YFP-KNL1$^{\Delta PP1}$	This study	N/A
pcDNA5-YFP-KNL1$^{PP1(2A)}$	This study	N/A
pcDNA5-YFP-KNL1 $^{\Delta NT}$	This study	N/A
pcDNA5-YFP-B56γ_1-KNL1$^{\Delta NT}$	This study	N/A
pcDNA5-YFP-B56 γ_1^{CD}-KNL1$^{\Delta NT}$	This study	N/A
pcDNA5-YFP-KNL1 $^{\Delta NT\text{-}PP1mid}$	This study	N/A
pcDNA5-YFP-BUBR1$^{\Delta CT\text{-}PP1:KNL1}$	This study	N/A
pcDNA5-YFP-BUBR1$^{\Delta CT\text{-}KNL1}$	This study	N/A
pcDNA5-YFP-BUBR1$^{\Delta CT}$-B56γ_1	This study	N/A
pMESV$_\Psi$-mCherry-B56γ_1	This study	N/A
pcDNA3-2xRFP-PP1γ^{WT}	Nijenhuis et al., 2014	N/A
pcDNA4-mTurquoise2-BUBR1^{WT}	This study	N/A
pcDNA5-mTurquoise2-BUBR1 $^{deP\text{-}PP2A}$	This study	N/A
pcDNA5-mTurquoise2-BUBR1$^{\Delta CT}$-B56γ_1	This study	N/A
pcDNA4-mTurquoise2-BUBR1$^{\Delta CT}$	This study	N/A
pcDNA4-mTurquoise2-BUBR1^{Long}	This study	N/A
pcDNA4-mTurquoise2-BUBR1$^{\Delta CT\text{-}PP1:KNL1}$	This study	N/A
pcDNA4-mTurquoise2-BUBR1$^{\Delta CT\text{-}KNL1}$	This study	N/A
pcDNA5-YFP-B56γ_1	Vallardi et al., 2019	N/A
pcDNA5-YFP-B56γ_1^{CD}	Vallardi et al., 2019	N/A
pOG44 Flp-Recombinase expression vector	Thermo Fisher	Cat# V600520
Software and algorithms		
GraphPad Prism 6	GraphPad Software	N/A
Fiji	Schindelin et al., 2012	https://imagej.net/Fiji
Micro-Manager	Edelstein et al., 2010	https://micro-manager.org/
Sloppycell	Myers et al., 2007	http://sloppycell.sourceforge.net/

LEAD CONTACT AND MATERIALS AVAILABILITY

Further information and requests for resources and reagents should be directed to and will be fulfilled by the Lead Contact, Adrian Saurin (a.saurin@dundee.ac.uk).

EXPERIMENTAL MODEL AND SUBJECT DETAILS

All cell lines were derived from HeLa Flp-in cells (Sex = female; a gift from S Taylor, University of Manchester, UK) (Tighe et al., 2008), which were authenticated by STR profiling (Eurofins). The cells were cultured in DMEM supplemented with 9% FBS and 50 μg/ml penicillin/streptomycin. During fluorescence time-lapse analysis, cells were cultured in Leibovitz's L-15 media (900 mg/L D+ Galactose, 5mM Sodium Pyruvate, no phenol red) or DMEM (no phenol red) supplemented with 9% FBS and 50μg/ml penicillin/streptomycin. Cells were screened every 4-8 weeks to ensure they were mycoplasma free.

METHOD DETAILS

Plasmids and cloning

pcDNA5-YFP-BUBR1^{WT} expressing an N-terminally YFP-tagged and siRNA-resistant wild-type BUBR1 and pcDNA5-YFP-BUBR1$^{\Delta PP2A}$ (also called BUBR1$^{\Delta KARD}$), lacking amino acids 663-680 were described previously (Nijenhuis et al., 2014). All the remaining YFP-BUBR1 mutants were subcloned by PCR amplification of DNA fragments followed by a Gibson assembly reaction to insert back into the original vector (pcDNA5-YFP-BUBR1^{WT}), except when indicated. pcDNA5-YFP-BUBR1 $^{\Delta CT}$, was subcloned from pcDNA5-YFP-BUBR1^{WT} by PCR amplification of the BUBR1 fragment (excluding amino acids 664-1050). pcDNA5-YFP-BUBR1^{Long} was constructed by insertion of a flexible 36 amino acid [GSG]-linker between amino acids 663 and 664. pcDNA5-YFP-BUBR1$^{deP\text{-}PP2A}$ was generated by site directed mutagenesis of the pcDNA5-YFP-BUBR1^{WT} vector mutating the KARD motif from 5′-SIKKLSPIIEDSR-3′ to 5′-RSSTLPTIHEEEE-3′ (Kruse et al., 2018).

pcDNA5-YFP-KNL1^{WT} expressing an N-terminally YFP-tagged and siRNA-resistant wild-type KNL1, pcDNA5-YFP-KNL1$^{PP1(2A)}$ (with S25A and S60A mutations, also called KNL1^{2SA}) and pcDNA5-YFP-KNL1$^{\Delta PP1}$ (with RVSF at amino acids 58-61 mutated to AAAA, also called KNL1^{4A}) were described previously (Nijenhuis et al., 2014). Site directed mutagenesis was used to further improve the resistance of pcDNA5-YFP-KNL1^{WT} construct to dsiRNA by modifying two extra codons (dsiRNA-resistant site 5′-GCACGT GAGCTTGAAGGAA-3′, nucleotides 2678-2676). While confirming the accuracy of the mutagenesis by sequencing, we detected a deletion of amino acids 910 to 1120 in the KNL1 constructs used previously, probably caused by Nijenhuis et al. (2014). This occurred between identical MELT13/17 sequences and was probably caused by recombination of the plasmid during bacterial culture. To correct this, a 3991 bp fragment (corresponding to nucleotides 2730-6720 in KNL1^{WT} plasmid) was amplified from genomic DNA of RPE cells and subcloned into the pcDNA5-YFP-KNL1^{WT} plasmid already containing the improved siRNA-resistant site. Gibson assembly was then performed to correct all KNL1 constructs by replacing the N terminus through XhoI and PmlI restriction sites using pcDNA5-YFP-KNL1$^{PP1(2A)}$ and pcDNA5-YFP-KNL1$^{\Delta PP1}$ as PCR templates. pcDNA5-YFP-KNL1 $^{\Delta NT}$ (with deletion of the first 70 amino acids of KNL1) was also created by Gibson assembly using the same restriction sites.

pcDNA5-YFP-B56γ_1-KNL1$^{\Delta NT}$ (KNL1 $^{\Delta NT}$ fused to B56γ_1) and pcDNA5-YFP-B56$\gamma_1{}^{CD}$-KNL1$^{\Delta NT}$ (KNL1 $^{\Delta NT}$ fused to a version of B56γ_1 with a S296D mutation that disrupts PP2A binding (Vallardi et al., 2019)) were produced by restriction cloning using fragments generated by PCR from pcDNA5-YFP-B56γ_1 and pcDNA5-YFP-B56$\gamma_1{}^{CD}$, respectively (Vallardi et al., 2019), both were inserted using NotI and KasI restriction sites creating a 28 amino acid linker before KNL1 $^{\Delta NT}$. To create pcDNA5-YFP-KNL1 $^{\Delta NT\text{-}PP1mid}$ a fragment containing the PP1-binding SILK and RVSF motifs (amino acids 24 to 70 of KNL1^{WT}) was inserted at a BlpI site of pcDNA5-YFP-KNL1 $^{\Delta NT}$ by Gibson assembly (between MELT-10 and MELT-11). pcDNA5-YFP-BUBR1$^{\Delta CT\text{-}PP1:KNL1}$ was created by Gibson assembly of two PCR fragments: pcDNA5-YFP-BUBR1 $^{\Delta CT}$ (amplified from pcDNA5-YFP-BUBR1^{WT}) and a fragment containing the first 70 amino acids of the N-terminal tail of KNL1 with the Aurora B sites mutated in the SLiM (amplified from pcDNA5-YFP-KNL1$^{PP1(2A)}$) (a 6 amino acid linker connects BUBR1$^{\Delta CT}$ to the N-terminal tail of KNL1). The same subcloning strategy was used to create pcDNA5-YFP-BUBR1$^{\Delta CT\text{-}KNL1}$ but using a mutated N-terminal tail of KNL1 which cannot recruit PP1 (amplified from pcDNA5-YFP-KNL1$^{\Delta PP1}$). pcDNA5-YFP-BUBR1$^{\Delta CT}$-B56γ_1 was also subcloned with the same strategy but using a fragment containing B56 amplified from pcDNA5-YFP-B56γ_1 (Vallardi et al., 2019), inserting B56γ_1 and a 7 amino acid linker after amino acid 658 of BUBR1.

pcDNA4-mTurquoise2(Turq2)-BUBR1^{WT} was created by Gibson assembly of 3 PCR fragments: pcDNA4 backbone, a fragment containing BUBR1^{WT} (amplified from pcDNA5-YFP- BUBR1^{WT}) and a fragment containing mTurquoise2. pcDNA5-Turq2-BUBR1 $^{deP\text{-}PP2A}$ was created by restriction cloning using Acc65I and BstBI to replace the YFP originally present in pcDNA5-YFP-BUBR1 $^{deP\text{-}PP2A}$ (Turq2 subcloned from pcDNA4-Turq2-BUBR1^{WT}). Similarly, Turq2-tagged version of BUBR1$^{\Delta CT}$-B56γ_1 was created by restriction cloning using NheI and NotI to remove YFP. A pcDNA4-Turq2 version of BUBR1$^{\Delta CT}$ was created by restriction cloning using BstBI and HpaI to replace BUBR1^{WT} from the original pcDNA4-Turq2-BUBR1^{WT} vector. In the same way, pcDNA4-Turq2-BUBR1^{Long} was created by restriction cloning using BstBI and Bsu36I, and pcDNA4-Turq2 versions of BUBR1$^{\Delta CT\text{-}PP1:KNL1}$ and BUBR1$^{\Delta CT\text{-}KNL1}$ were created by restriction cloning using BstBI and NotI. pMESV$_\Psi$-mCherry-B56γ_1 was produced by Gibson assembly of pMESV$_\Psi$-mCherry backbone (amplified from pMESV$_\Psi$-mCherry-CenpB-Mad1 (Maldonado and Kapoor, 2011)) and

B56γ_1 (amplified from pcDNA5-YFP-B56γ_1) (Vallardi et al., 2019). pcDNA3-2xRFP-PP1 γ was cloned previously (Nijenhuis et al., 2014). All plasmids were fully sequenced to verify the transgene was correct.

Gene expression

HeLa Flp-in cells stably expressing doxycycline-inducible constructs were derived from the HeLa Flp-in cell line by transfection with the relevant pcDNA5/FRT/TO vector and the Flp recombinase pOG44 (Thermo Fisher). Cells were subsequently selected in media containing 200 μg/ml hygromycin B (Santa Cruz biotechnology) for at least 2 weeks to select for stable integrants at the FRT locus. Cells expressing mCherry-B56γ_1 in combination with YFP-tagged KNL1$^{WT/\Delta PP1}$ were generated by viral-integration of pMESV$_\Psi$-mCherry-B56γ_1 construct into the genome of HeLa Flp-in cells, followed by puromycin selection (1μg/ml, Santa Cruz biotechnology) and were then transfected as above with YFP-KNL1$^{WT/\Delta PP1}$. Double mutant analysis was performed using cells that express a combination of Turq2-tagged BUBR1 and YFP-tagged KNL1. These were generated by transient transfection of Turq2-tagged constructs into cells that were stably expressing doxycycline-inducible YFP-tagged recombinant proteins (generated as described above). These Turq2-tagged constructs were transfected 32 hours prior to endogenous gene knock-down (described below) and at least 72 hours prior to imaging or fixation. PP1γ localization was examined using RFP-tagged PP1γ transiently transfect into YFP-BUBR1 cell lines 32 hours prior to knockdown/replacement of BUBR1. Plasmids were transfected into HeLa Flp-in cells using Fugene HD (Promega) according to the manufacturer's instructions.

Gene knockdown

For all experiments involving re-expression of BUBR1 or KNL1 in HeLa Flp-in cells, the endogenous mRNA was knocked down and replaced with an siRNA-resistant mutant. To knockdown endogenous BUBR1 or KNL1 or both together, cells were transfected with 20nM BUBR1 siRNA (5′- AGAUCCUGGCUAACUGUUC –3′) (Sigma-Aldrich) or 20nM KNL1 dsiRNA (sense: 5′-GCAUGUAUCU CUUAAGGAAGAUGAA-3′; antisense: 5′-UUCAUCUUCCUUAAGAGAUACAUGCAU-3′) (Integrated DNA technologies) or both simultaneously for 16 h after which the cells were arrested in early S phase by addition of thymidine (2mM, Sigma-Aldrich) for 24 h. All siRNAs were transfected using Lipofectamine® RNAiMAX Transfection Reagent (Life Technologies) according to the manufacturer's instructions. BUBR1 and KNL1 construct expression was induced by the addition of doxycycline (1μg/ml, Sigma-Aldrich) during and following the thymidine block. After thymidine block, cells were release into media supplemented with doxycycline and, where appropriate, nocodazole (3.3 μM, Sigma-Aldrich) for 5-7 hours for live imaging or 8.5 hours for fixed analysis. MPS1 and Aurora B were inhibited by adding AZ-3146 (Sigma-Aldrich) and/or ZM-447439 (Cayman Chemicals) shortly prior to live cell imaging. For kinase inhibition in cells analyzed by immunofluorescence, nocodazole and MG132 (10 μM, Sigma-Aldrich) were added first for 30 minutes, followed by a time-course of AZ-3146 and/or ZM-447439 with nocodazole and MG132.

Immunofluorescence

Cells, plated on High Precision 1.5H 12-mm coverslips (Marienfeld), were fixed with 4% paraformaldehyde (PFA) in PBS for 10 min or pre-extracted (when using pRVSF-KNL1 or mCherry antibodies) with 0.1% Triton X-100 in PEM (100 mM Pipes, pH 6.8, 1 mM MgCl2 and 5 mM EGTA) for 1 minute before addition of 4% PFA for 10 minutes. Coverslips were washed with PBS and blocked with 3% BSA in PBS + 0.5% Triton X-100 for 30 min, incubated with primary antibodies overnight at 4°C, washed with PBS and incubated with secondary antibodies plus DAPI (4,6- diamidino2-phenylindole, Thermo Fisher) for an additional 2-4 hours at room temperature in the dark. Coverslips were then washed with PBS and mounted on glass slides using ProLong antifade reagent (Molecular Probes). All images were acquired on a DeltaVision Core or Elite system equipped with a heated 37°C chamber, with a 100x/1.40 NA U Plan S Apochromat objective using softWoRx software (Applied precision). Images were acquired at 1x1 binning using a CoolSNAP HQ or HQ2 camera (Photometrics) and processed using softWorx software and ImageJ (National Institutes of Health). All immunofluorescence images displayed are maximum intensity projections of deconvolved stacks and were chosen to most closely represent the mean quantified data.

Chromosome alignment assays

To observe chromosome alignment, cells were released from thymidine block for 7 hours before being synchronized at the G2/M boundary with a 2-hour treatment with RO-3306 (10 μM, Tocris). Cells were then washed three times and incubated for 15 minutes with full growth media before addition of MG132 for 30 minutes to prevent mitotic exit. Cells were then fixed and stained as described above and scored based on the number of misaligned chromosomes as aligned (0 misaligned chromosomes), mild (1-2), moderate (3-5) or severe (> 6). This protocol is important because mutants that cause a prolonged arrest can otherwise cause cohesion fatigue, which skews the alignment data.

Time-lapse analyses

For fluorescence imaging, cells were imaged in 8-well chamber slides (ibidi) in Leibovitz L-15 media with a heated 37°C chamber or in DMEM (no phenol red) with a heated 37°C chamber in 5% CO_2. Images were taken every 4 minutes with either a 20x /0.4 NA air objective using a Zeiss Axio Observer 7 with a CMOS Orca flash 4.0 camera at 4x4 binning or a 40x/1.3 NA oil objective using a DV Elite system equipped with Photometrics CascadeII:1024 EMCCD camera at 4x4 binning. For brightfield imaging, cells were imaged in a 24-well plate in DMEM in a heated chamber (37°C and 5% CO_2) with a 10x/0.5 NA objective using a Hamamatsu ORCA-ER camera at 2x2 binning on a Zeiss Axiovert 200M, controlled by Micro-manager software (open source: https://micro-manager.org/) or with a

20x/0.4 NA air objective using a Zeiss Axio Observer 7 as detailed above. Mitotic exit was defined by cells flattening down in the presence of nocodazole and MPS1 inhibitor. In assays where both recombinant BUBR1 and KNL1 are expressed in cells, cells were selected for quantification based on high levels of Turq2 as an indication of successful transient transfection of Turq2-BUBR1 constructs into cells stably expressing YFP-tagged KNL1 constructs.

Antibodies

All antibodies were diluted in 3% BSA in PBS. The following primary antibodies were used for immunofluorescence imaging (at the final concentration indicated): chicken α-GFP (ab13970 from Abcam, 1:5000), mouse α-GFP (clone 4E12/8, a gift from P. Parker, 1:1000), rabbit α-pNDC80 Serine 55 (GTX70017 from GeneTex, 1:1000), guinea pig α-Cenp-C (BT20278 from Caltag + Medsystems, 1:5000), rabbit α -BUB1 (A300-373A from Bethyl, 1:1000), mouse α-BUBR1 (A300-373A from Millipore, 1:1000), rabbit α-mCherry (GTX128508, Genetex,1:1000). The rabbit α-pMELT-KNL1 antibody is directed against Thr 943 and Thr 1155 of human KNL1 (Nijenhuis et al., 2014) (Gift from G.Kops, Hubrecht, NL). The pRVSF-KNL1 (pSer60-KNL1) antibody (custom rabbit polyclonals, gift from I. Cheeseman, MIT, USA) was used at 1:2000 dilution (Nijenhuis et al., 2014). Secondary antibodies used were highly-cross absorbed goat, α-chicken, α-rabbit, α-mouse or a-guinea pig coupled to Alexa Fluor 488, Alexa Fluor 568, or Alexa Fluor 647 (Thermo Fisher).

Mathematical modeling approach, terminology and assumptions

The model consists of a set of ordinary differential equations (ODEs) that correspond to the diagram in Figure 5A. All binding/dissociation and phosphorylation/dephosphorylation reactions were modeled according to simple mass-action kinetics.

In the following, "[X]" stands for the amount of species X. If X is an 'atomic' species (i.e., corresponding to one of the individual rectangular boxes in Figure 5A), then "$[X_{tot}]$" denotes the total amount of species X, including phosphorylated forms and all complexes that contain X as a component, while "$[X_{free}]$" stands for the amount of X that is unbound and unphosphorylated. "pX" stands for the phosphorylated form of X and "X : Y" for a form in which species X and Y are bound. Binding/dissociation rates for species X are referred to as "kb_X" and "kd_X" and phosphorylation/dephosphorylation rates as "kp_X" and "kdp_X," respectively.

Using ODEs means that the behavior of individual molecules is not represented and that all species are assumed to be well-mixed. Thus, effectively the model describes the processes occurring on all KNL1 molecules located on one representative kinetochore. We assume that the total amounts for all species are conserved, except for MPS1 and Aurora B whose amounts can be changed externally to simulate the experimental inhibition of kinases. Furthermore, we assume that BUBR1 can be phosphorylated both in its free form and when bound to pMELT but allow that this may happen with different rates (corresponding to the parameters $kp_{BUBR1(free)}$ and kp_{BUBR1}). PP1 and PP2A are assumed to be catalytically active only when bound to KNL1 (via RVSF and pMELT:pBUBR1, respectively). In reality, PP2A can also bind to unphosphorylated BUBR1, but we neglect this possibility because phos- phorylation by CDK1 and PLK1 increases the binding affinity by 11-fold and 5-fold, respectively (Wang et al., 2016a). To keep the model as simple as possible we assumed that one phosphorylation event (CDK1) is needed to allow PP2A binding. For brevity, the active phosphatases are denoted by "$PP1_{act}$" and "$PP2_{act}$."

The assumption of no substrate specificity means that PP1 and PP2A act on the same substrates (pMELT, pRVSF, pMELT: pBUBR1, and pNDC80) and that for each substrate there is only one parameter describing the catalytic activities of both.

Modeling equations

The model can be described using a set of nine equations:

$$\frac{d[PP1_{act}]}{dt} = kb_{PP1} \cdot [RVSF] \cdot [PP1_{free}] - kd_{PP1}[PP1_{act}]$$

$$\frac{d[PP2A_{act}]}{dt} = kb_{PP2A} \cdot [pMELT : pBUBR1] \cdot [PP2A_{free}] + kb_{BUBR1}[pBUBR1 : PP2A] \cdot [pMELT] - kd_{PP2A} \cdot [PP2A_{act}] - kd_{BUBR1} \cdot [PP2A_{act}]$$

$$\begin{aligned}\frac{d[pBUBR1_{free}]}{dt} = {} & kd_{BUBR1} \cdot [pMELT : BUBR1] + kd_{PP2A}[pBUBR1 : PP2A] + kp_{BUBR1(free)} \cdot [BUBR1_{free}] \cdot [CDK1] \\ & - kb_{BUBR1} \cdot [BUBR1_{free}] \cdot [pMELT] - kb_{PP2A} \cdot [pBUBR1_{free}] \cdot [PP2A_{free}]\end{aligned}$$

$$\begin{aligned}\frac{d[pBUBR1 : PP2A]}{dt} = {} & kb_{PP2A} \cdot [pBUBR1_{free}] \cdot [PP2A_{free}] + kd_{BUBR1} \cdot [PP2A_{act}] - kd_{PP2A} \cdot [pBUBR1 : PP2A] \\ & - kb_{BUBR1} \cdot [pBUBR1 : PP2A] \cdot [pMELT]\end{aligned}$$

$$\begin{aligned}\frac{d[pMELT]}{dt} = {} & kd_{BUBR1} \cdot [pMELT : BUBR1] + kd_{BUBR1} \cdot [pMELT : pBUBR1] + kd_{BUBR1} \cdot [PP2A_{act}] + kp_{MELT} \cdot [MELT] \cdot [MPS1] \\ & - kb_{BUBR1} \cdot [BUBR1_{free}] \cdot [pMELT] - kb_{BUBR1} \cdot [pBUBR1_{free}] \cdot [pMELT] - kb_{BUBR1} \cdot [pBUBR1 : PP2A] \cdot [pMELT] \\ & - kdp_{MELT} \cdot [pMELT] \cdot ([PP1_{act}] + [PP2A_{act}])\end{aligned}$$

$$\frac{d[\mathrm{pMELT:BUBR1}]}{dt} = kb_{\mathrm{BUBR1}} \cdot [\mathrm{BUBR1}_{\mathrm{free}}] \cdot [\mathrm{pMELT}] + kdp_{\mathrm{BUBR1}} \cdot [\mathrm{pMELT:pBUBR1}] \cdot ([\mathrm{PP1}_{\mathrm{act}}] + [\mathrm{PP2A}_{\mathrm{act}}]) - kd_{\mathrm{BUBR1}} \cdot [\mathrm{pMELT:BUBR1}] - kp_{\mathrm{BUBR1}} \cdot [\mathrm{pMELT:BUBR1}] \cdot [\mathrm{CDK1}]$$

$$\frac{d[\mathrm{pMELT:pBUBR1}]}{dt} = kb_{\mathrm{BUBR1}} \cdot [\mathrm{pBUBR1}_{\mathrm{free}}] \cdot [\mathrm{pMELT}] + kp_{\mathrm{BUBR1}} \cdot [\mathrm{pMELT:BUBR1}] \cdot [\mathrm{CDK1}] + kd_{\mathrm{PP2A}} \cdot [\mathrm{PP2A}_{\mathrm{act}}] - kd_{\mathrm{BUBR1}} \cdot [\mathrm{pMELT:pBUBR1}] - kdp_{\mathrm{BUBR1}} \cdot [\mathrm{pMELT:pBUBR1}] \cdot ([\mathrm{PP1}_{\mathrm{act}}] + [\mathrm{PP2A}_{\mathrm{act}}]) - kb_{\mathrm{PP2A}} \cdot [\mathrm{pMELT:pBUBR1}] \cdot [\mathrm{PP2A}_{\mathrm{free}}]$$

$$\frac{d[\mathrm{pRVSF}]}{dt} = kp_{\mathrm{RVSF}} \cdot [\mathrm{RVSF}] \cdot [\mathrm{AurB}] - kdp_{\mathrm{RVSF}} \cdot [\mathrm{pRVSF}] \cdot ([\mathrm{PP1}_{\mathrm{act}}] + [\mathrm{PP2A}_{\mathrm{act}}])$$

$$\frac{d[\mathrm{pNDC80}]}{dt} = kp_{\mathrm{NDC80}} \cdot [\mathrm{NDC80}] \cdot [\mathrm{AurB}] - kdp_{\mathrm{NDC80}} \cdot [\mathrm{pNDC80}] \cdot ([\mathrm{PP1}_{\mathrm{act}}] + [\mathrm{PP2A}_{\mathrm{act}}])$$

Free amounts and total amounts for the modeling

The free amounts can be determined by the following algebraic relations, given that the total amounts are conserved:

$$[\mathrm{PP1}_{\mathrm{free}}] = [\mathrm{PP1}_{\mathrm{tot}}] - [\mathrm{PP1}_{\mathrm{act}}]$$

$$[\mathrm{PP2A}_{\mathrm{free}}] = [\mathrm{PP2A}_{\mathrm{tot}}] - [\mathrm{PP2A}_{\mathrm{act}}] - [\mathrm{pBUBR1:PP2A}] - [\mathrm{BUBR1:PP2A}]$$

$$[\mathrm{BUBR1}_{\mathrm{free}}] = [\mathrm{BUBR1}_{\mathrm{tot}}] - [\mathrm{pBUBR1}_{\mathrm{free}}] - [\mathrm{pMELT:BUBR1}] - [\mathrm{pMELT:pBUBR1}] - [\mathrm{pBUBR1:PP2A}] - [\mathrm{BUBR1:PP2A}] - [\mathrm{PP2A}_{\mathrm{act}}]$$

$$[\mathrm{MELT}_{\mathrm{free}}] = [\mathrm{MELT}_{\mathrm{tot}}] - [\mathrm{pMELT}] - [\mathrm{pMELT:BUBR1}] - [\mathrm{pMELT:pBUBR1}] - [\mathrm{PP2A}_{\mathrm{act}}]$$

$$[\mathrm{RVSF}_{\mathrm{free}}] = [\mathrm{RVSF}_{\mathrm{tot}}] - [\mathrm{pRVSF}] - [\mathrm{PP1}_{\mathrm{act}}]$$

$$[\mathrm{NDC80}_{\mathrm{free}}] = [\mathrm{NDC80}_{\mathrm{tot}}] - [\mathrm{pNDC80}]$$

In order to compare the model results to the experimental measurements, we defined the total amount of phosphorylated MELT as an auxiliary variable in the following way:

$$[\mathrm{pMELT}_{\mathrm{tot}}] = [\mathrm{pMELT}] + [\mathrm{pMELT:BUBR1}] + [\mathrm{pMELT:pBUBR1}] + [\mathrm{PP2A}_{\mathrm{act}}]$$

The fixed total amounts were chosen according to the following assumptions: First, there are multiple MELT motifs, but only one RVSF site on each KNL1 molecule. Second, the species that bind to these motifs are available in sufficient amounts to potentially 'saturate' the motifs. Finally, total amounts for PP1 and PP2A are equal.

The choice of units is arbitrary because we only used relative amounts when comparing the model output to experimental data, and for clarity we leave out all units in the following. The values for the external kinases (MPS1, Aurora B, and CDK1) can be chosen arbitrarily without loss of generality because their activities are separately determined by fitting the respective parameters.

Given these assumptions, we chose the following values:

Species	Amount
$[\mathrm{PP1}_{\mathrm{tot}}]$	10
$[\mathrm{PP2A}_{\mathrm{tot}}]$	10
$[\mathrm{MELT}_{\mathrm{tot}}]$	10
$[\mathrm{BUBR1}_{\mathrm{tot}}]$	10
$[\mathrm{RSVF}_{\mathrm{tot}}]$	1
$[\mathrm{NDC80}_{\mathrm{tot}}]$	1
[MPS1]	1
[Aurb]	1
[CDK1]	1

Implementation of mutants and parameter optimization

The remaining parameters were determined by fitting simulated time courses of $[pMELT_{tot}]$ to two pieces of data. The first were the experimental data from Figure 1c-f. More specifically, we started the simulations from the steady state corresponding to a condition of metaphase arrest ($[MPS1] = [AurB] = 1$) and set either $[MPS1] = 0$ or $[MPS1] = [AurB] = 0$. The WT condition corresponds to the model as defined above, the ΔPP2A and ΔPP1 mutants were implemented by setting $[PP2A_{tot}]$ and $[PP1tot] = 0$, respectively. Initial amounts for $[pMELT_{tot}]$ were fit to be ≈50% of $[MELT_{tot}]$ in WT, ≈80% in ΔPP2A, and ≈100% in ΔPP1. To be able to investigate the contribution of negative feedback in a more fine-grained way, parameters were additionally fit to the data in Figure S5. Here, we considered $[PP2A_{act}]$ as a readout for B56γ. The B56γ^{CD} mutant was implemented by replacing all occurrences of the sum $[PP1_{act}] + [PP2A_{act}]$ in the above equations by $[PP1_{act}]$.

Based on these data, we obtained the following parameter set:

Parameter	Value
kb_{PP1}	0.13
kd_{PP1}	0.20
kb_{PP2A}	0.32
kd_{PP2A}	1.86
kb_{BUBR1}	0.18
kd_{BUBR1}	14.21
kp_{BUBR1}	0.076
$kp_{BUBR1(free)}$	0.0076
kdp_{BUBR1}	3.35
kp_{MELT}	0.37
kdp_{MELT}	0.70
kp_{RVSF}	72.91
kdp_{RVSF}	11.95

For Figure 5E the BUBR1-PP2A mutant was implemented by assuming that BUBR1 phosphorylation and PP2A binding are quasi-instantaneous and irreversible (setting $kp_{BUBR1} = kp_{BUBR1\ (free)} = kb_{PP2A} = 10.000$ and $kdp_{BUBR1} = kd_{PP2A} = 0$). The KNL1-PP2A mutant was implemented by setting $kb_{PP2A} = kd_{PP2A} = 0$and using a fixed amount of $[PP2A_{act}] = 1$, which corresponds to one copy of PP2A per molecule of KNL1. The curves in Figure 5E were generated by calculating the steady state value of $[pMELT_{tot}]$ for different levels of $[MPS1]$. Sensitivity is commonly defined as the slope in a logarithmic plot (Ferrell and Ha, 2014).

Computational methods

The model was implemented in the Systems Biology Markup Language (SBML) and is available in Data S1. Parameter fitting and simulations were performed using the Python package SloppyCell (Myers et al., 2007) and further analyzed using custom scripts written in Python.

Motif analysis

A set of PP1-binding peptides for the RVxF-binding pocket and PP2A-B56-binding peptides for the LxxIxE-binding pocket were created from experimentally validated peptides in the PP1 and PP2A literature. The dataset contained 110 RVxF and 27 LxxIxE motifs. A position-specific scoring matrix (PSSM) was constructed from each set of peptides based on amino acid frequencies weighted using peptide similarity weights and pseudocounts using the PSI BLAST IC scoring scheme as defined in the PSSMSearch tool (Altschul et al., 1997; Krystkowiak et al., 2018). Each PSSM was screened against the human UniProt reviewed proteins using PSSMSearch (Krystkowiak et al., 2018) and filtered using PSSM score *p value* with a cut-off of 0.0001, taxonomic range based on conservation of the consensus outside the mammalian clade, localization based on intracellular localization GO terms, and accessibility based on: (i) overlap with a resolved region in a structure from PDB, (ii) intrinsic disorder predictions (retaining only peptides found in disordered regions as defined by an IUPred score < 0.3 [15769473]) and (iii) UniProt annotation of topologically inaccessible regions (e.g., transmembrane and extracellular regions) [25348405]. Applying these criteria, we produced sets of predicted 344 RVxF-binding and 210 PP2A-B56-binding motifs (Table S1). The phosphorylated (experimentally validated phosphorylation sites annotated in the UniProt, phospho.ELM or phosphosite databases) or phosphorylatable (any serine or threonine) residues within the predicted and validated sets were collected and the kinase specificity of each site was annotated as basophilic ([KR]xS or [KR]xxS), acidophilic ([DEN]x[ST]) or proline-directed ([ST]P). Enrichment of motif specificity determinants were calculated as the binomial probability ($prob^{aa} = binomial(k,n,p)$) where k is the observed residue count at each position for a residue, n is the number of the instances of motifs and p is the background amino acid frequency of a residue based on the disordered regions of the human

proteome. Enrichment of groupings (KR - basic, DE - acidic, ST - phosphorylatable by serine/threonine kinases) was calculated similarly. RVxF-binding motifs are available at http://slim.ucd.ie/motifs/pp1/index.php?page=instances and PP2A-B56-binding motifs are available at http://slim.ucd.ie/pp2a/index.php?page=instances

QUANTIFICATION AND STATISTICAL ANALYSIS

For quantification of immunostainings, all images of similarly stained experiments were acquired with identical illumination settings and cells expressing comparable levels of exogenous protein were selected and analyzed using ImageJ. An ImageJ macro was used to threshold and select all kinetochores and all chromosome areas (excluding kinetochores) using the DAPI and anti-kinetochore antibody channels, as previously (Saurin et al., 2011). This was used to calculate the relative mean kinetochore intensity of various proteins ([kinetochores-chromosome arm intensity (test protein)] / [kinetochores-chromosome arm intensity (CENP-C)]. To quantify RFP-PP1γ levels, a 10 × 0.2 μM z stack projection of kinetochore rich z sections were used. Following imaging, any cells that were not in focus or out of view were excluded from the analysis.

Two-tailed, unpaired t tests with Welch's correction were performed to compare the means values between experimental groups in immunofluorescence quantifications (using Prism 6 software). The raw data for all experiments and the actual statistical values can be found in Data S2.

DATA AND CODE AVAILABILITY

The published article contains all the datasets generated in Data S2 and the code for the model data is available in SBML format in Data S1.

Cell Reports, Volume *28*

Supplemental Information

PP1 and PP2A Use Opposite Phospho-dependencies

to Control Distinct Processes at the Kinetochore

Richard J. Smith, Marilia H. Cordeiro, Norman E. Davey, Giulia Vallardi, Andrea Ciliberto, Fridolin Gross, and Adrian T. Saurin

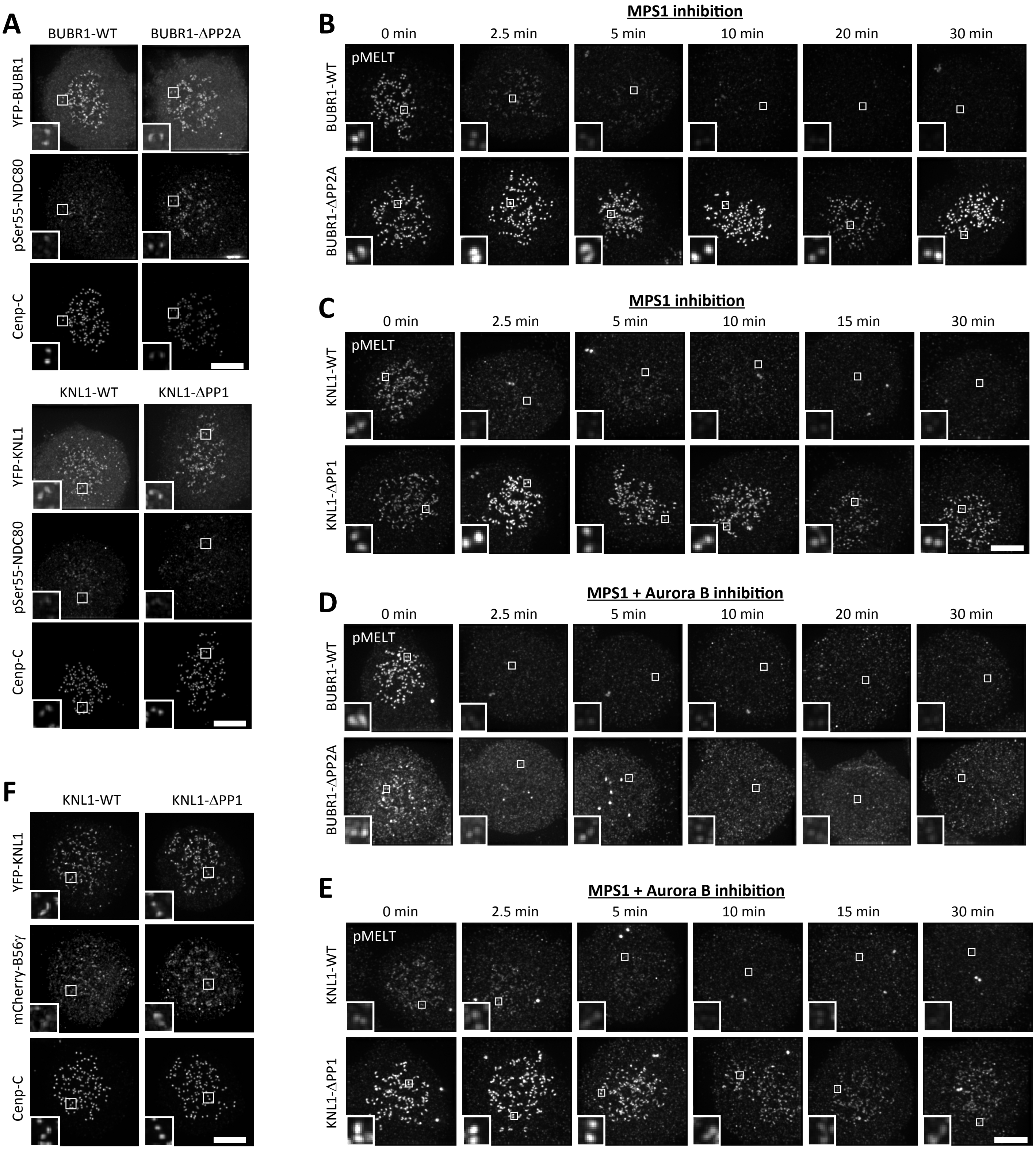

Figure S1. Immunofluorescence images to show that PP1-KNL1 and PP2A-B56 exert control over different pathways and processes at the kinetochore; related to figure 1. A-F. Example immunofluorescence images of the kinetochore quantifications shown in Figure 1B (A), 1C (B), 1D (C), 1E (D), 1F (E) and 1H (F). The images were chosen that most closely resemble the mean values in the quantifications. The insets show magnifications of the outlined regions. Scale bars = 5μm.

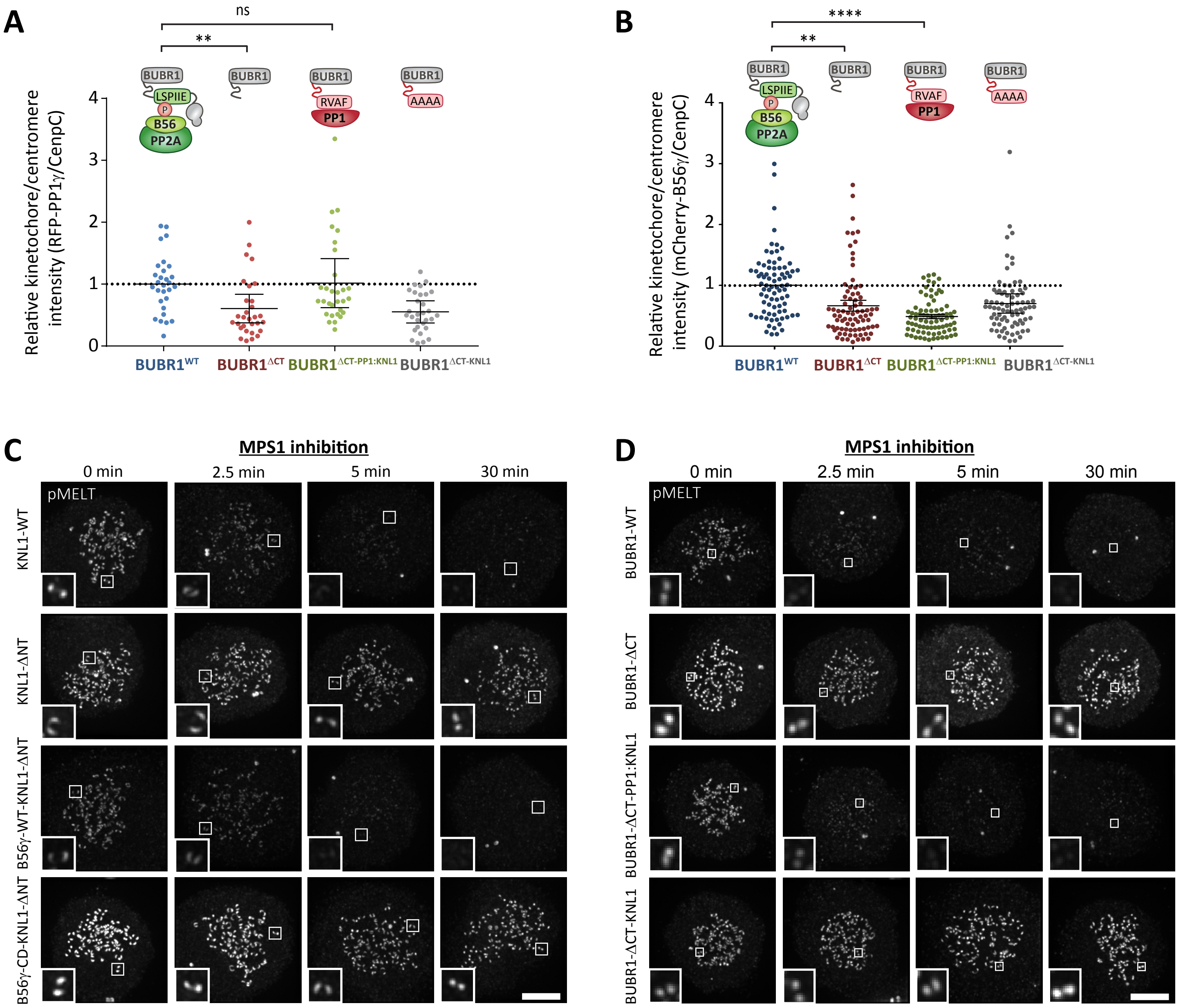

Figure S2. Quantification of kinetochore phosphatase levels and immunofluorescence images after swapping the phosphatase recruitment SLiMs between BUBR1 and KNL1; related to figure 2. A. RFP-PP1γ levels in the various mutant BUBR1 cells lines. Note that full removal of KNL1-PP1 from kinetochores only causes a 70% reduction in total kinetochore PP1γ (Nijenhuis et al., 2014). Graph shows 30 cells per condition from 3 experiments. **B.** Quantification of kinetochore mCherry-B56γ levels in the various mutant BUBR1 cells lines. Note that removal of the B56-SLiM on BUBR1 does not fully remove B56γ because a pool of B56γ remains bound to the centromere via Sgo1 (Vallardi et al., 2019) and this pool is also detected in our quantifications because this does not distinguish between kinetochores and centromeres. Graph shows 80 cells per condition from 4 experiments. For all kinetochore intensity graphs, each dot represents a cell and the errors bars display the variation between the experimental repeats (displayed as ±SD of the experimental means). **$p<0.01$, **** $p<0.0001$. **C-D.** Example immunofluorescence images of the kinetochore quantifications shown in Figure 2B (A) and 2D (B). The images were chosen that most closely resemble the mean values in the quantifications. The insets show magnifications of the outlined regions. Scale bars = 5µm.

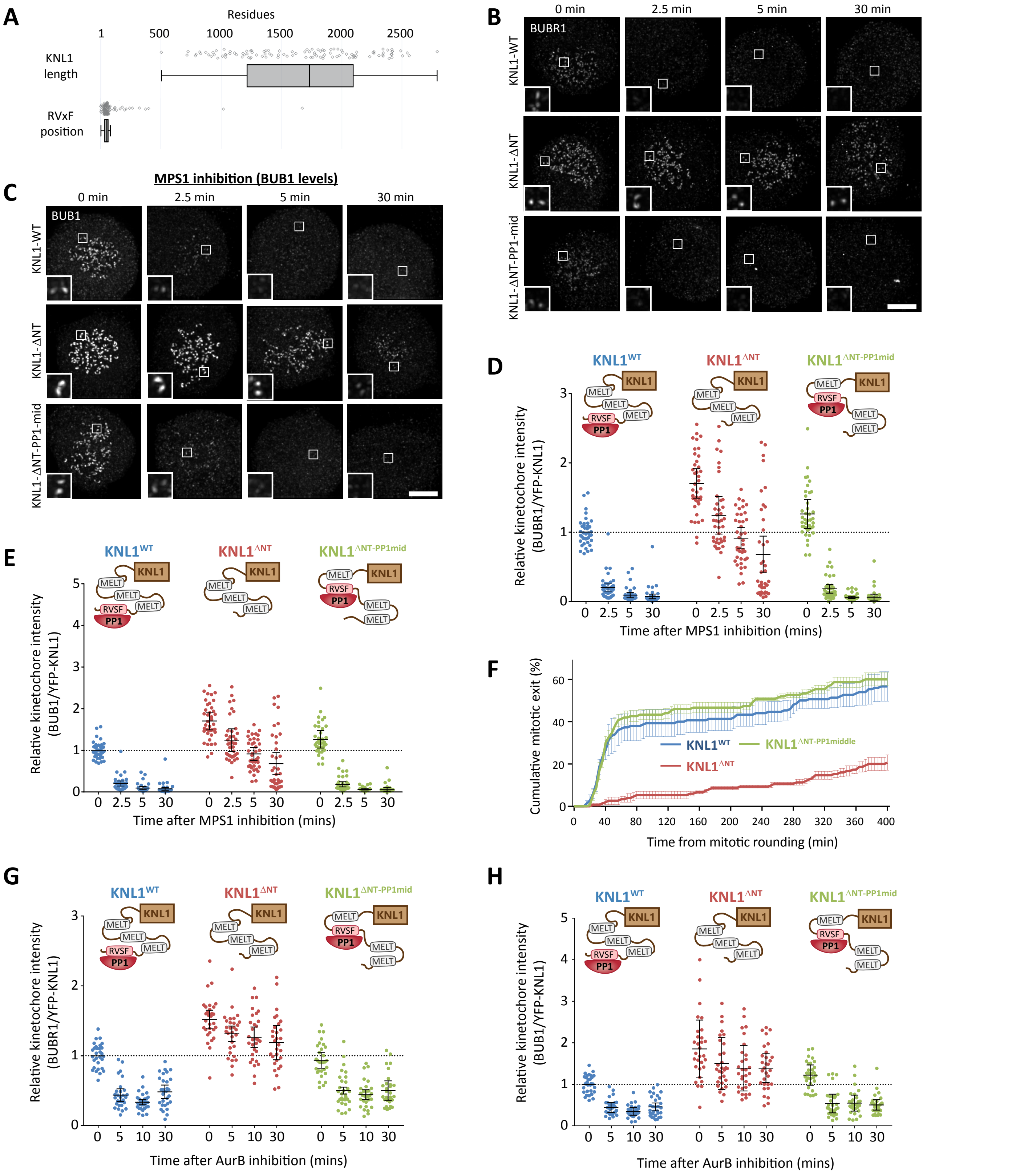

Figure S3. The PP1 binding SLiM is conserved at the N-terminus of KNL1 and this position is not critical for MELT dephosphorylation in nocodazole; related to figure 3. A. Position of first RVxF motif and total number of amino acids in 110 KNL1 orthologs from all eukaryotic supergroups; as identified in (Tromer et al., 2015). **B-E.** Example immunofluorescence images (B,C) and quantifications of kinetochore intensities (D,E) of BUBR1 (B,D) and BUB1 (C,E) from cells treated identically to those described in Figure 3C. **F.** Duration of mitotic arrest in cells treated with nocodazole and 2.5 µM AZ-3146. Graph shows the cumulative mean (±SEM) of 3 experiments, 50 cells per condition per experiment. **G,H.** Quantifications of kinetochore intensities of BUBR1 (G) and BUB1 (H) from nocodazole-arrested cells treated with the Aurora B inhibitor ZM-447439 (2 µM) for the indicated times. Kinetochore intensities graphs show 40 cells per condition from 4 experiments (D,E) or 30 cells from 3 experiments (G,H). MG132 was included to prevent Cyclin B degradation and mitotic exit following MPS1 inhibition. Each dot represents a cell and the errors bars display the variation between the experimental repeats (displayed as ±SD of the experimental means). The images were chosen that most closely resemble the mean values in the quantifications. The insets show magnifications of the outlined regions. Scale bars = 5µm.

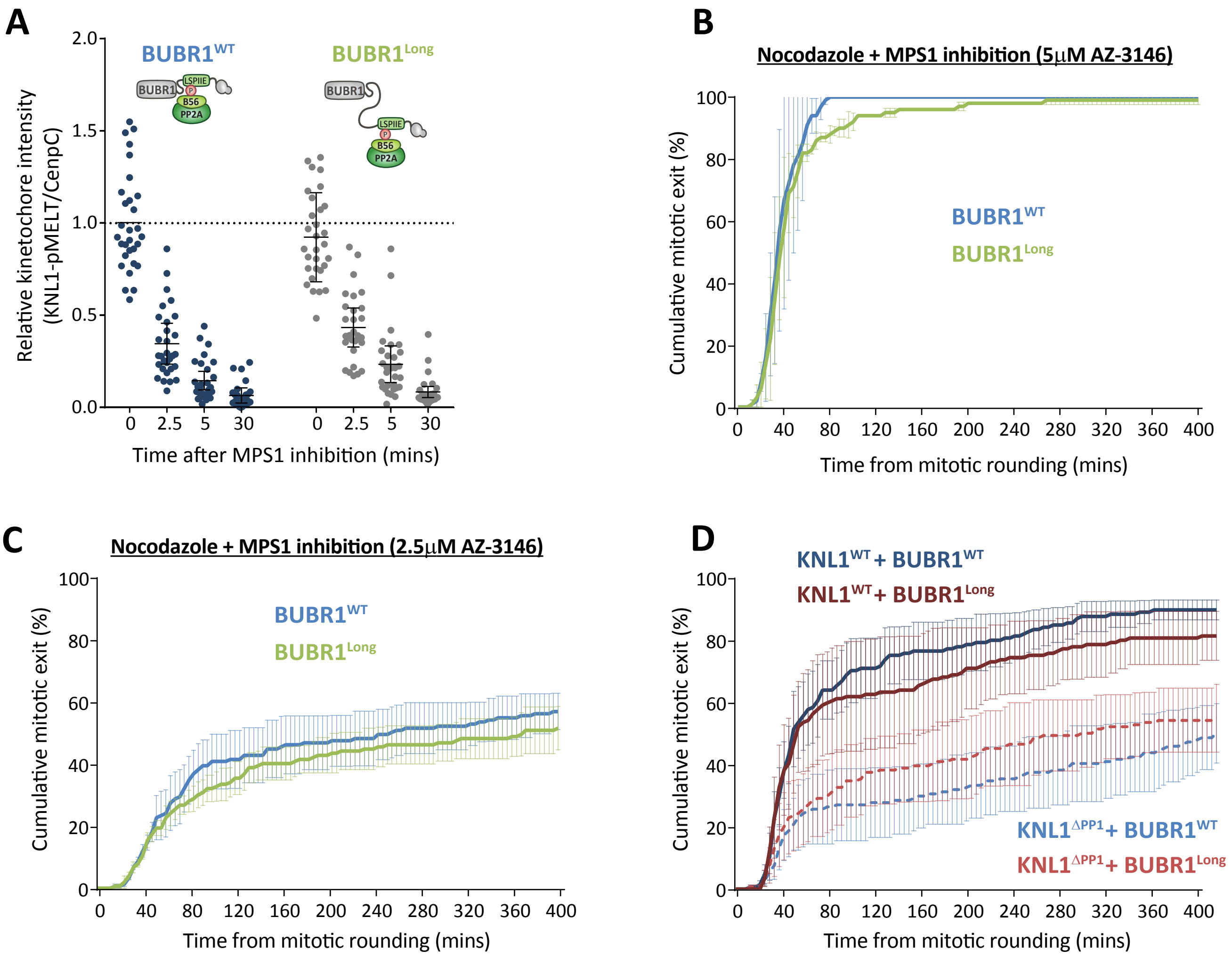

Figure S4. Insertion of a long flexible linker before the LxxIxE motif in BUBR1 does not improve the ability of PP2A to silence the SAC; related to figure 4. Effect of inserting a long flexible linker before the LxxIxE motif in BUBR1 on SAC phenotypes. **A.** KNL1-MELT dephosphorylation in nocodazole-arrested cells treated with 2.5 µM AZ-3146 for indicated times. MG132 was included in to prevent Cyclin B degradation and mitotic exit following MPS1 inhibition. Graph shows 30 cells per condition from 3 experiments. Each dot represents a cell and the errors bars display the variation between the experimental repeats (displayed as ±SD of the experimental means). **B-D.** Duration of mitotic arrest in cells treated with nocodazole and 5 µM (B) or 2.5 µM (C,D) AZ-3146. Experiments in D contain wild type and mutant combinations to remove PP1-KNL1 in the presence or absence of the flexible linker. Graph in B-D show the cumulative mean (±SEM) of 3 experiments, 50 cells per condition per experiment. 2 experiments from the BubR1-WT controls in D are also used in some experiments from Figure 3E (See Data File S2 for details).

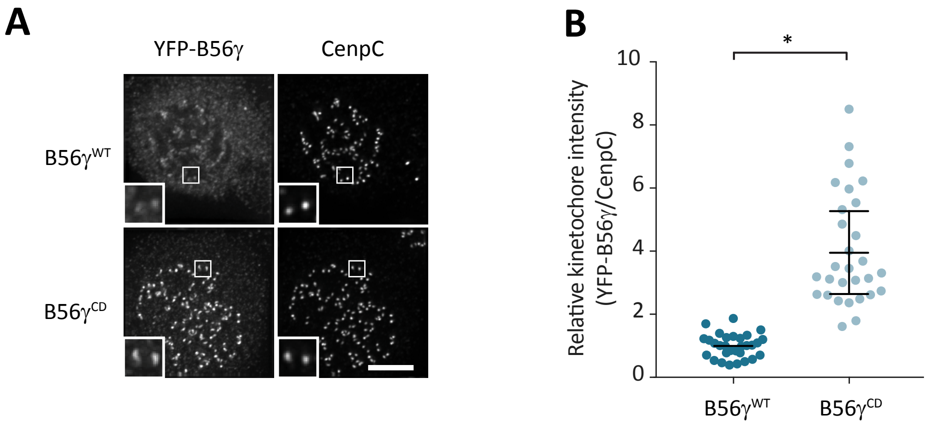

Figure S5. Effect of negative feedback on B56γ kinetochore levels; related to figure 5. A,B. Example immunofluorescence images (A) and quantification of kinetochore intensities (B) of YFP-B56γWT or YFP-B56γCD. The images were chosen that most closely resemble the mean values in the quantifications. The insets show magnifications of the outlined regions. Scale bars = 5μm. ns $p>0.05$, $*p<0.05$.

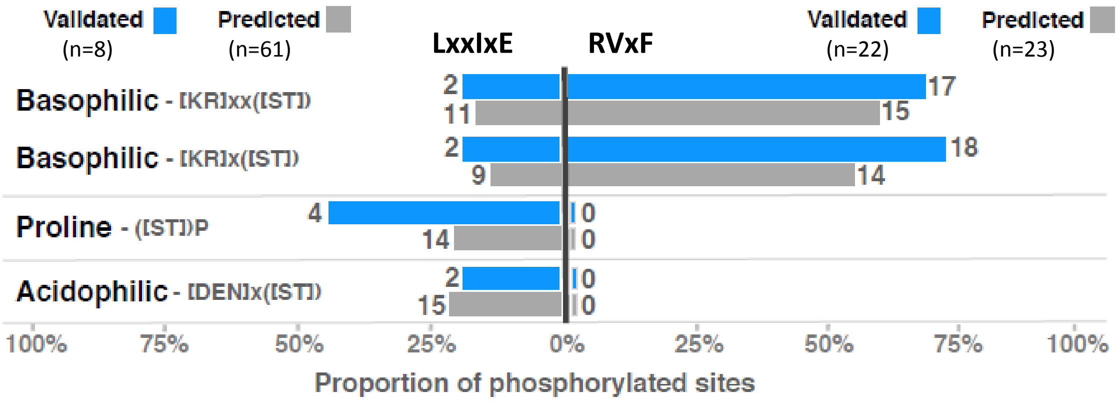

Figure S6. Summary of the kinase specificity determinants overlapping the phosphorylated and phosphorylatable sites in the experimentally validated LxxIxE and RVxF motifs; related to figure 6. The kinase specificity determinants of proline-directed, basophilic and acidophilic kinases matching the preferences of Cyclin dependent kinases, Aurora kinases and the Polo-like kinases, respectively. Note that Aurora phosphorylation of CDK1 sites is prevented, even if the correct basic sequences are present, because proline (+1) is a strong negative determinant within the Aurora consensus motifs (Alexander et al., 2011).
